# MEGF11 is related to tumour recurrence in triple negative breast cancer via chemokine upregulation

**DOI:** 10.1038/s41598-020-64950-0

**Published:** 2020-05-15

**Authors:** Jen-Hwey Chiu, Ling-Ming Tseng, Tzu-Ting Huang, Chun-Yu Liu, Jir-You Wang, Ching-Po Huang, Yi-Fang Tsai, Chih-Yi Hsu

**Affiliations:** 1 Comprehensive Breast Health Center & Division of General Surgery, Department of Surgery, Taipei Veterans General Hospital, Taipei, Taiwan, ROC; 2 Institute of Traditional Medicine, School of Medicine, National Yang-Ming University, Taipei, Taiwan, ROC; 3 Department of Surgery, Cheng-Hsin General Hospital, Taipei, Taiwan, ROC; 4 Department of Surgery, School of Medicine, National Yang-Ming University, Taipei, Taiwan, ROC; 5 Women’s Malignancies Branch, Center for Cancer Research, National Cancer Institute, Bethesda, MD USA; 6 Division of Medical Oncology, Department of Oncology, Taipei Veterans General Hospital, Taipei, Taiwan, ROC; 7 Department of Orthopedics, Taipei-Veterans General Hospital, Taipei, Taiwan, ROC; 8 Faculty of Medicine, School of Medicine, National Yang-Ming University, Taipei, Taiwan, ROC; 9 Institute of Clinical Medicine, School of Medicine, National Yang-Ming University, Taipei, Taiwan, ROC; 10 Department of Pathology and Laboratory Medicine, Taipei Veterans General Hospital, Taipei, Taiwan, ROC; 11 School of Medicine, National Yang-Ming University, Taipei, Taiwan, ROC

**Keywords:** Breast cancer, Metastasis

## Abstract

Our previous study demonstrated that upregulation of multiple epidermal growth factor-like domains 11 (MEGF11) gene expression is involved in the mechanism by which recurrence of Triple Negative Breast Cancer (TNBC) occurs. Our aim was to elucidate the role of MEGF11 expression in TNBC cells, both *in vitro* and *in vivo*, and in human tissue. Following MEGF11 gene knockdown (∆*MEGF11*) or over-expression in MDA-MB-231 and MB-468 cells, cell growth and chemokine gene expression were evaluated. *In vivo*, tumour growth of implanted human TNBC cells and the number of circulating 4T1 mouse tumour cells were measured. There was a significant decrease in cell growth via inhibition of AKT, NF-kB, CREB and AP-1 activation in ∆*MEGF11* MDA-MB-231 and 468 cells. This also resulted, *in vivo*, in a suppression of tumour growth and a decrease in the number of mouse circulating 4T1 breast cancer cells. Surprisingly, overexpression of MEGF11 upregulated the expression of various chemokines and proinflammatory cytokines via AKT activation, but there was no increase in cell proliferation. MEGF11 was found to cross-talk positively with IL-17A signalling. Patients with tumours that over-expressed MEGF11 had a poorer prognosis. We conclude that MEGF11 plays an important role in tumour survival and that overexpression of MEGF11 induces both a cytokine and a chemokine cascade, which will favour the tumour microenvironment in terms of distant metastasis. MEGF11 might be a potential therapeutic target for preventing TNBC recurrence.

## Introduction

Breast cancer is the most common invasive female cancer worldwide^1,2^. Triple negative breast cancer (TNBC) is characterized by its occurrence in younger women, its aggressive tumour behaviour, and a high association with metastasis to distant organs. TNBC shows strong resistance to hormonal therapy, chemotherapy, and targeted therapy^3–5^. Many biomarkers associated specifically with TNBC subtypes have been identified^6–9^ and several targeted therapies, such as receptor tyrosine kinase and SRC family inhibitors, have been investigated in clinical trials^10^. Nevertheless, the results have been of limited usefulness when treating TNBC patients.

The epidermal growth factor (EGF)-like domain, a highly conserved protein domain, has been found in many animal proteins^11^. Based on the different functions found to be associated with these multiple EGF like domains, distinct domain subtypes have been identified^12^. Previous investigations have shown that EGF-like domains play important roles in immune responses^13^, apoptosis^14^, and calcium binding^15,16^. Recently, much effort has been devoted to studying the correlation between the multiple epidermal growth factor-like domain (MEGF) subtypes and their functions. For example, MEGF10 is postulated to act as a tumour repressor gene in neuroblastoma^17^, while genetic aberrations affecting MEGF10 are associated with myopathy^18,19^, areflexia, respiratory distress and dysphagia (EMARDD)^20^. Mutations in MEGF8 have been shown to be highly associated with the Carpenter syndrome subtype that shows defective left-right patterning^21^. Nonetheless, the functions of many MEGF subtypes, such as MEGF6, MEGF7, MEGF9, and MEGF11, still remain to be elucidated.

Up to the present, few reports have investigated the role of MEGF11 in mammalian species, although this protein does share a substantial similarity with MEGF10 and they are likely to represent a novel protein family^22,23^. Recent evidence has demonstrated that MEGF10 and MEGF11 play a crucial role in the formation of mosaics through two retinal interneuron subtypes, namely, starburst amacrine cells and horizontal cells^24^. However, information concerning the role of MEGF11 in cancer, specifically TNBC, is lacking.

A recent bioinformatics study that targeted the molecular mechanisms present in TNBC tumours demonstrated that several genes were differentially expressed in paired tumour samples when recurrent and non-recurrent patients were compared^25^. In addition to the above, cDNA open array analysis of 224 genes using paired TNBC tissue samples (16 recurrent and 24 non-recurrent tissues) showed that *MEGF11* was significantly upregulated in tumour tissues from patients where there was subsequent clinical recurrence compared to patients without recurrence. Accordingly, the aim of the present study was to elucidate the role of MEGF11 in human TNBC cells, *in vitro*, *in vivo* and in human patients.

## Results

### Identification of the role of *MEGF11* in recurrent TNBC

**Figure 1:**
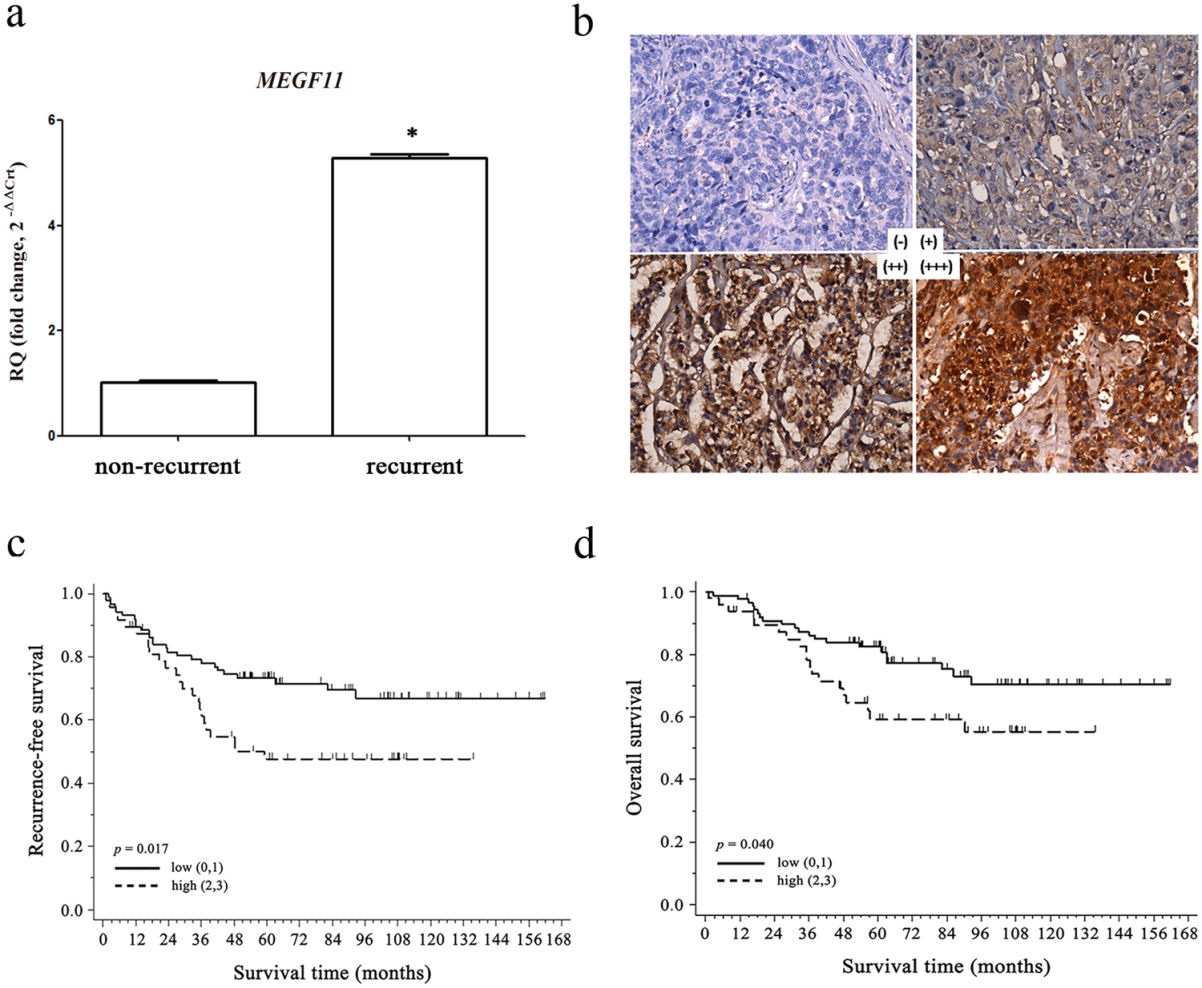
Identification of *MEGF11* in recurrent triple negative breast cancer. Using cDNA open array chips, 224 genes in paired TNBC tissue samples (16 recurrent and 24 non-recurrent tissues) were analysed, and *MEGF11* was significantly upregulated in tumour tissues with subsequent clinical recurrence compared with those without recurrence (**a**). Protein expression by immunohistochemistry (**b**) was correlated with patient survival, including recurrence-free survival (**c**) and overall survival (**d**). The protein expression of MEGF11 was semi-quantified and expressed as (0), <10%, (1), 11–25%, (2), 26–50%, and (3)>50% of tumour cells. The MEGF11 expression level was defined as low (≤25%, n = 87) and high (> 25%, n = 48). Data are presented as the mean ± SD. Asterisks indicate a p value <0.05 by Mann-Whitney U test, and Kaplan-Meier survival analysis was performed with Prism 5 software.

To investigate the critical genes associated with the recurrence of TNBC, we conducted a cDNA open array analysis involving 224 expressed genes using paired TNBC tissue samples (16 recurrent and 24 non-recurrent patients) (Supplementary Information 1) and found that *MEGF11* was significantly upregulated in tumour tissue that was associated with subsequent clinical recurrence compared to those without recurrence (Fig. 1a). Kaplan-Meier plots demonstrated that there was a significant negative correlation between MEGF11 protein expression level (Fig. 1b) and recurrence-free survival (RFS) (Fig. 1c) and overall survival (OS) (Fig. 1d). In addition, the results of the Kaplan-Meier plotter database indicated that patients split by the upper quartile also showed a negative correlation between *MEGF11* gene upregulation and patient RFS (Supplementary Information 2).

### Knockdown of *MEGF11* in the two TNBC cell lines decreased cell proliferation via suppression of the AKT, mTOR and NF-κB signalling pathways

**Figure 2:**
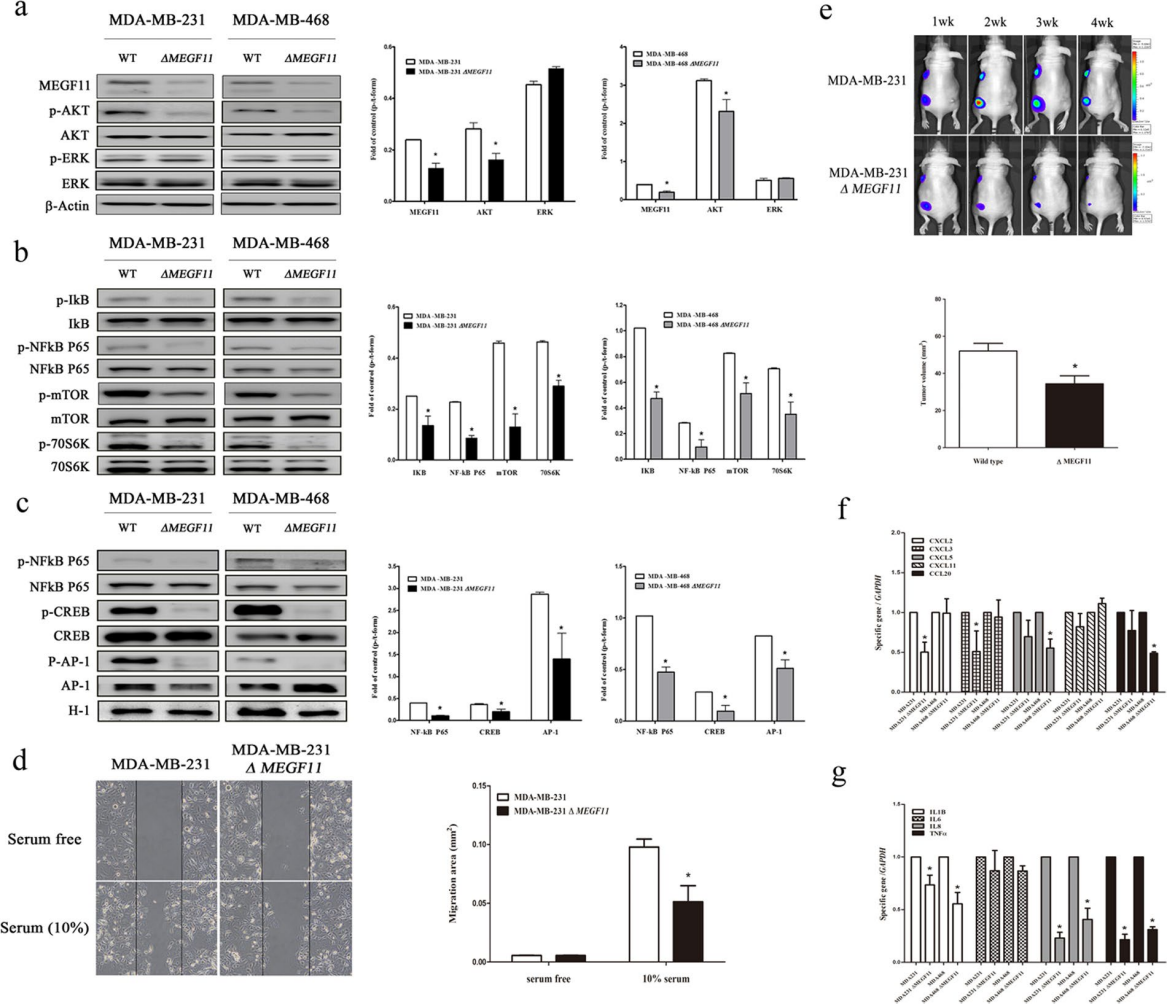
Knocked down *MEGF11* in TNBC cell lines decreased cell proliferation through suppression of AKT, mTOR and NF-κB signalling pathways. *MEGF11* was knocked down with short hairpin RNA (shRNA) in the TNBC cell lines MDA-MB-231 and MDA-MB-468. Cell proliferation-related signalling proteins including AKT, ERK (**a**), mTOR, and NF-κB (**b**) and nuclear factors NF-κB p65, CREB, and AP-1 (**c**) were analysed by Western blot (n = 4–6). Cell migration activity (**d**) and *in vivo* tumour growth rate (**e**) were evaluated by wound healing assay (n = 6) and an *in vivo* imaging system (IVIS) in nude mice (n = 6), respectively. The mRNA transcripts of chemokines including CCL20, CXCL2, CXCL5, and CXCL11(**f**) and cytokines including IL1β, TNF-α, IL6, and IL8 (**g**) were quantified with real-time PCR (n = 4–6). Asterisks indicate a p value <0.05 in Δ*MEGF11* TNBC cells compared to the wild type by Mann-Whitney U test.

To determine the roles of MEGF11 in tumour behaviour, we knocked down *MEGF11* in two TNBC cell lines, MDA-MB-231 and MDA-MB-468, and found that there was a significant decrease in the cell proliferation rates of both types of ∆*MEGF11* cells; the doubling times of the wild type MDA-MB-231 and MDA-MB-468 cells were 1.57 d and 2.54 d, respectively, and those of the ∆*MEGF11* MDA-MB-231 and ∆*MEGF11* MDA-MB-468 lines were 4.34 d and 3.25 d, respectively (Supplementary Information 3). Western blot analysis showed that knockdown of *MEGF11* significantly affected AKT (Fig. 2a), mTOR and NF-κB signalling (Fig. 2b) and decreased the expression of various transcription factors, including NF-κB p65, CREB, and AP-1, in the nuclei of ∆*MEGF11* MDA-MB-231 and ∆*MEGF11* MDA-MB-468 cells (Fig. 2c). Furthermore, the cell migration (Fig. 2d) and *in vivo* growth rate (Fig. 2e) of the Δ*MEGF11* MDA-MB-231 cells were both significantly lower than those of the wild-type cells. It should also be noted that many chemokines, including CCL20, CXCL2, and CXCL5, as well as various cytokines, such as IL1β, TNF-α, and IL17-A, were downregulated by knocking down *MEGF11* in the two TNBC cell lines (Fig. 2f,g). These results suggested that MEGF11 played an important role in modulating cell proliferation and cytokine/chemokine production in TNBC cells.

### Over-expression of MEGF11 upregulates the gene expression of chemokines and proinflammatory cytokines via AKT activation but does not affect cell proliferation

**Figure 3:**
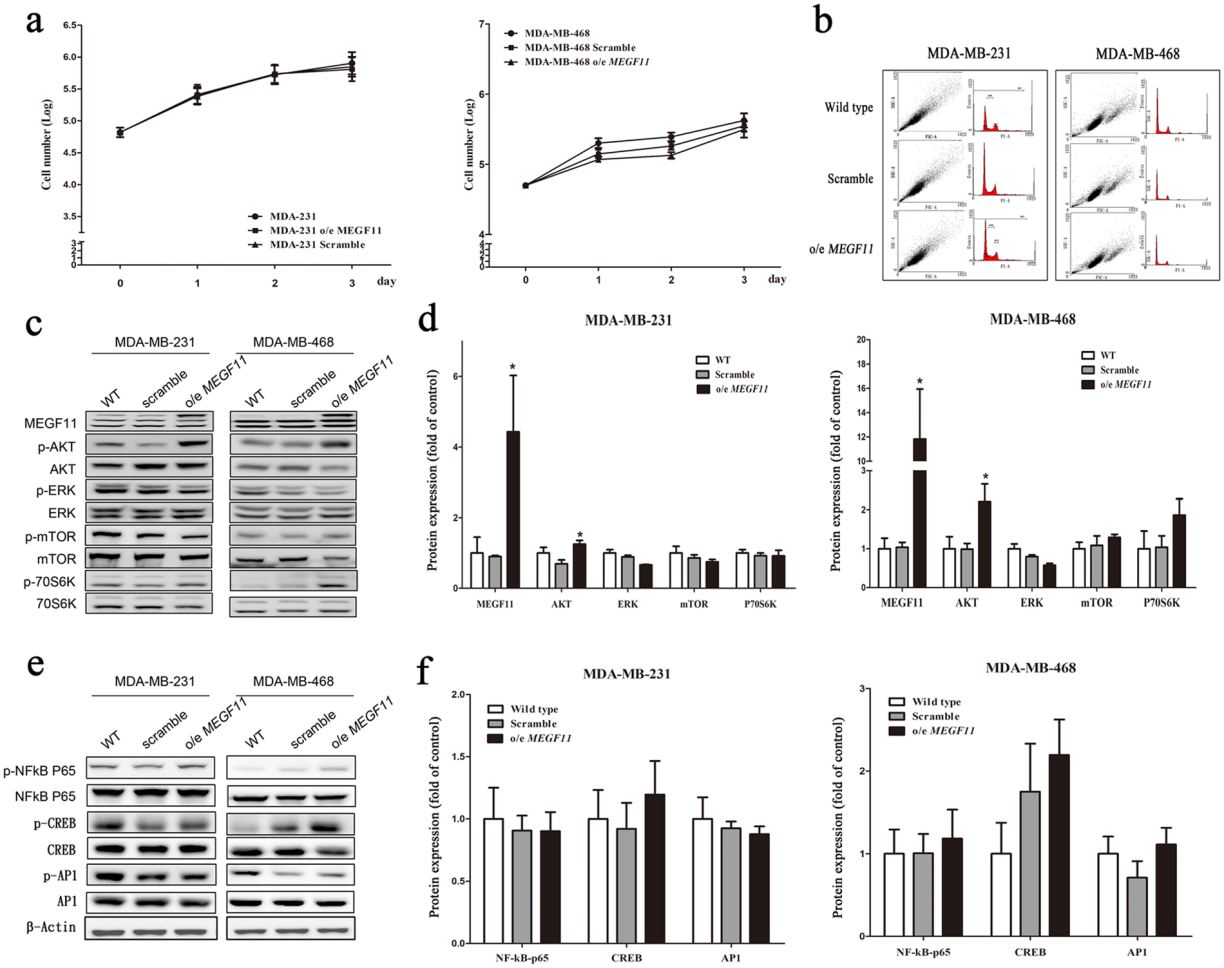
Over-expression of MEGF11 did not promote cell proliferation. The MEGF11 over-expression vector was cloned into the pCMV-AC-GFP vector. After MEGF11 was over-expressed in TNBC cells, the cell number (**a**) was evaluated by Trypan blue exclusion assay and verified by cell cycle analysis (**b**). Growth-related signalling proteins including AKT, ERK, mTOR, and p70s6K (**c**,**d**), and nuclear factors NF-κB, CREB, and AP-1 (**e**,**f**), were analysed by Western blot and quantified using wild type as a control group. The growth curves were analysed with two-way ANOVA. Asterisks indicate a p value <0.05 in o/e *MEGF11* TNBC cells compared to the scramble group by Mann-Whitney U test (n = 4).

**Figure 4:**
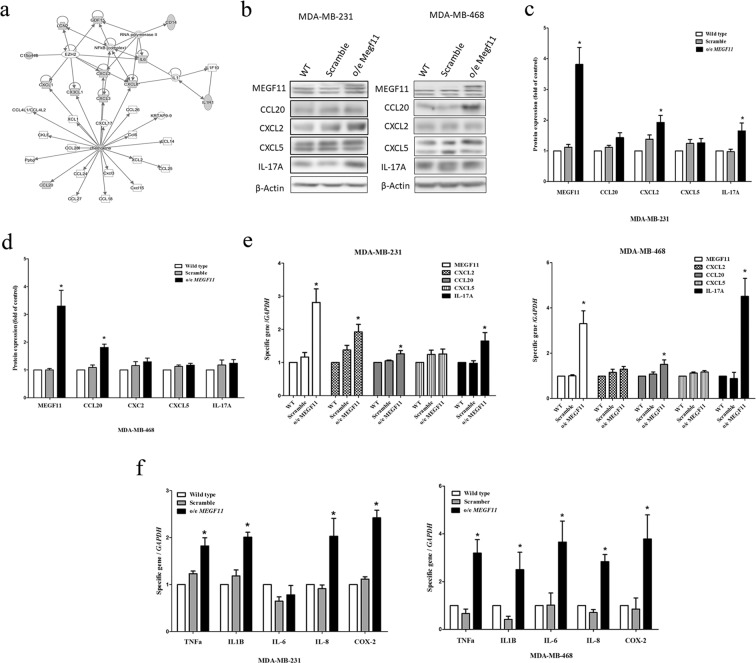
Over-expression of MEGF11 increased the upregulation of chemokines and proinflammatory cytokine gene expression. Following ingenuity pathway analysis (**a**), the expressions of chemokines including CCL20, CXCL2, CXCL5 and IL-17A in the o/e *MEGF11* MDA-MB-231 line (**b**,**c**) and MDA-MB-468 cells (**d**) were analysed by Western blot (n = 5) and quantified using wild type as a control group. The mRNA transcripts of chemokines including CCL20, CXCL2, CXCL5 (**e**), and cytokines including TNF-α, IL1β, IL-6, IL-8 and COX2 (**f**) were quantified with real-time PCR. Asterisks indicate a p value <0.05 in o/e *MEGF11* TNBC cells compared to the scramble group by Mann-Whitney U or Student’s t test (n = 3–4).

When MEGF11 was over-expressed in TNBC cells, the cell proliferation activity, in terms of both cell numbers (Fig. 3a), and cell cycle analysis (Fig. 3b, Supplementary Information 4), was not changed in either MDA-MB-231 or MDA-MB-468 compared to the MEGF11 wild type cells. When analysed by Western blotting, there was a significant increase in AKT activation, but there was no effect on ERK, mTOR, p70s6K (Fig. 3c,d), NF-κB, CREB, and AP-1 activation (Fig. 3e,f) using o/e MEGF11 TNBC cells compared to the scramble groups. In contrast, ingenuity pathway analysis indicated that MEGF11 plays a role in the chemokine and cytokine upregulation (Fig. 4a) (Supplementary Information 5). Western blotting (Fig. 4b) also demonstrated that there was an increase in the expression of CXCL2 and IL-17A, but not CCL20, CXCL5, in o/e MEGF11 MDA-MB-231 cells (Fig. 4c), but only of CCL20 in o/e MEGF11 MDA-MB-468 cells (Fig. 4d). There was also upregulation of the expression of CCL20, CXCL2 and IL-17A genes in o/e MEGF11 MDA-MB-231 cells, while upregulation of CCL20 and IL-17A gene expression in o/e MEGF11MDA-MB-468 cells (Fig. 4e). Besides, there was upregulation of various proinflammatory cytokines, such as *TNF-α*, *IL-1*β, *IL-8*, and *COX2* (Fig. 4f), in both types of TNBC cells over-expressing MEGF11, except that *IL-6* gene upreglation was noticed only in o/e MEGF11MDA-MB-468 cells.

### Cross-talk between MEGF11 and IL-17A

**Figure 5:**
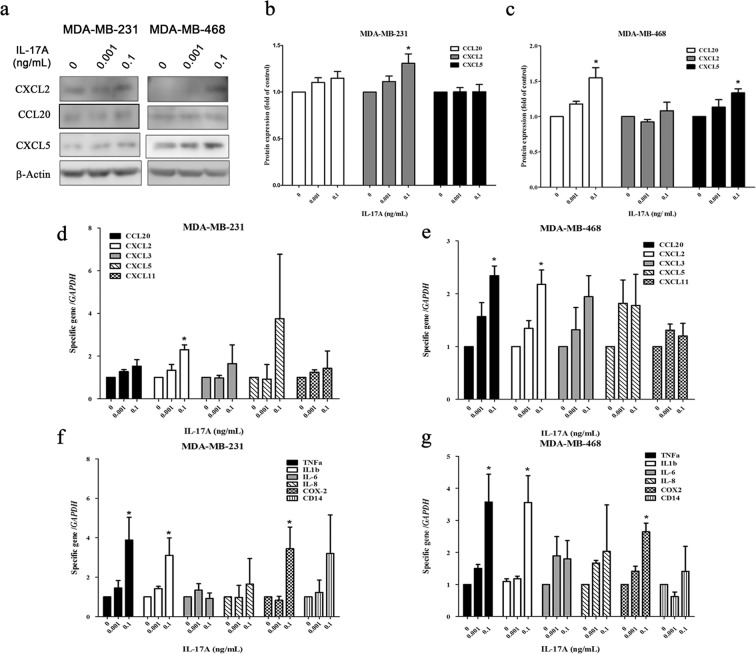
Cross-talk between MEGF11 and IL-17A. *MEGF11* was knocked down with short hairpin RNA (shRNA) and over-expressed with the pCMV-AC-GFP vector in TNBC cell line MDA-MB-231 and MDA-MB-468 cells. When the MEGF11 gene was knocked down, mRNA transcripts of IL-17A in Δ*MEGF11* (**a**) and o/e *MEGF11* (**b**) were quantified by real-time PCR (n = 4). After administration of different doses (0, 0.001, 0.1 ng/mL) of IL-17A in cultured media, MEGF11 protein (**c**) and IL-17A-related signalling proteins in MDA-MB-231 (**d**) and MDA-MB-468 (**e**) were analysed by Western blot (n = 4–6). The mRNA transcripts of *MEGF11*, *IL-17A*, and IL-17 receptors (*IL17RB*, *IL17RC*) in MDA-MB-231 and MDA-MB-468 cells (**f**) were quantified with real-time PCR (n = 6). Data between two groups were analysed with Mann-Whitney U or Student’s t test, while dose-related data were analysed with one-way ANOVA, followed by Dunnett’s post hoc test. Asterisks indicate a p value <0.05 compared to the wild type (for Δ*MEGF11*), the scramble group (for o/e *MEGF11*) or the vehicle group (for dose-dependent study).

When the MEGF11 gene was knocked down in the two TNBC cell lines, IL-17A transcripts (Fig. 5a) were found to be decreased in both cell lines. In contrast, there was increased IL-17A protein expression in o/e MDA-MB-231 cells (Fig. 4b,c) but not in o/e MDA-MB-468 cells (Fig. 4b,d). Furthermore, there were increased expression levels of IL-17A mRNA in both o/e MEGF11 lines (Fig. 5b). After the addition of IL-17A to the culture media, an increase in MEGF11 protein levels was observed (Fig. 5c). Src and ERK activation (Fig. 5d,e) and upregulation of IL-17A and IL-17RB genes, but not IL-17RC, were also observed in MDA-MB-231 and MDA-MB-468 cells (Fig. 5f). These results indicated that a degree of positive cross-talk between MEGF11 and IL-17A was occurring and that an IL-17A autocrinal loop affects TNBC cells.

### IL-17A upregulates the gene expressions of chemokines and proinflammatory cytokines

**Figure 6:**
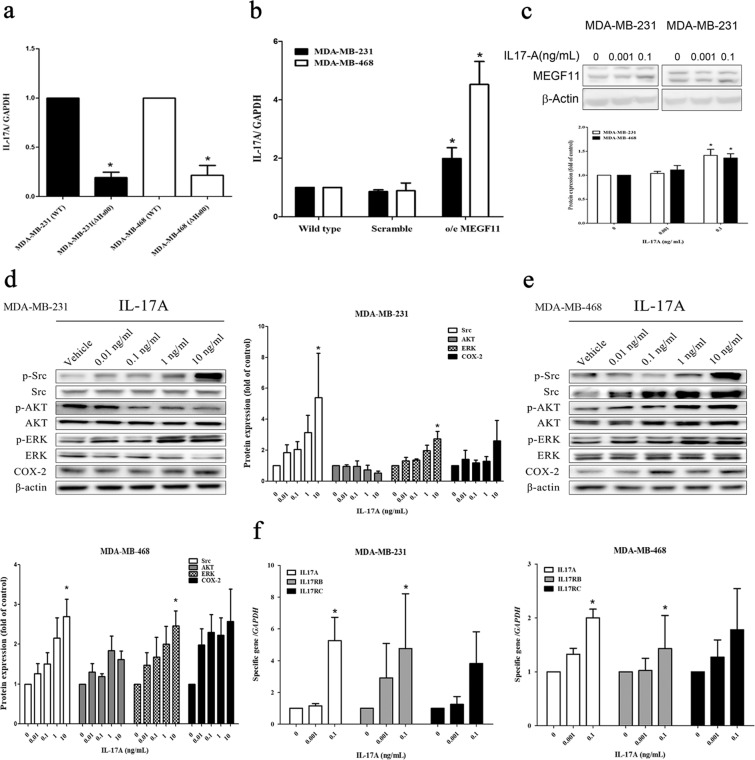
IL-17A increased the upregulation of chemokines and proinflammatory cytokine gene expression. After administration of different doses (0, 0.001, 0.1 ng/mL) of IL-17A in cultured media of MDA-MB-231 and MDA-MB-468 cells, chemokines including CCL20, CXCL2, and CXCL5 (**a**–**c**) were analysed by Western blot (n = 4). The mRNA transcripts of chemokines (**d**,**e**) and proinflammatory cytokines including TNF-α, IL-1β, IL-6, IL-8, and COX2 (**f**,**g**) were quantified with real-time PCR (n = 6). Data were analysed with one-way ANOVA, followed by Dunnett’s post hoc test. Asterisks indicate a p value <0.05 compared to the vehicle group.

In addition to increased MEGF11 protein expression, IL-17A also significantly increased CXCL2 and CCL20 expression in the MDA-MB-231 and MDA-MB-468 cell lines, respectively, both at the protein level (Fig. 6a–c) and at the mRNA level (Fig. 6d,e). Furthermore, IL-17A was able to upregulate expression in TNBC cells of various proinflammatory cytokines, such as TNF-α, IL-1β and COX2 (Fig. 6f,g).

### Knockdown of *MEGF11* in a mouse 4T1 mammary cancer cell line decreases tumour weight and the number of circulating tumour cells

**Figure 7:**
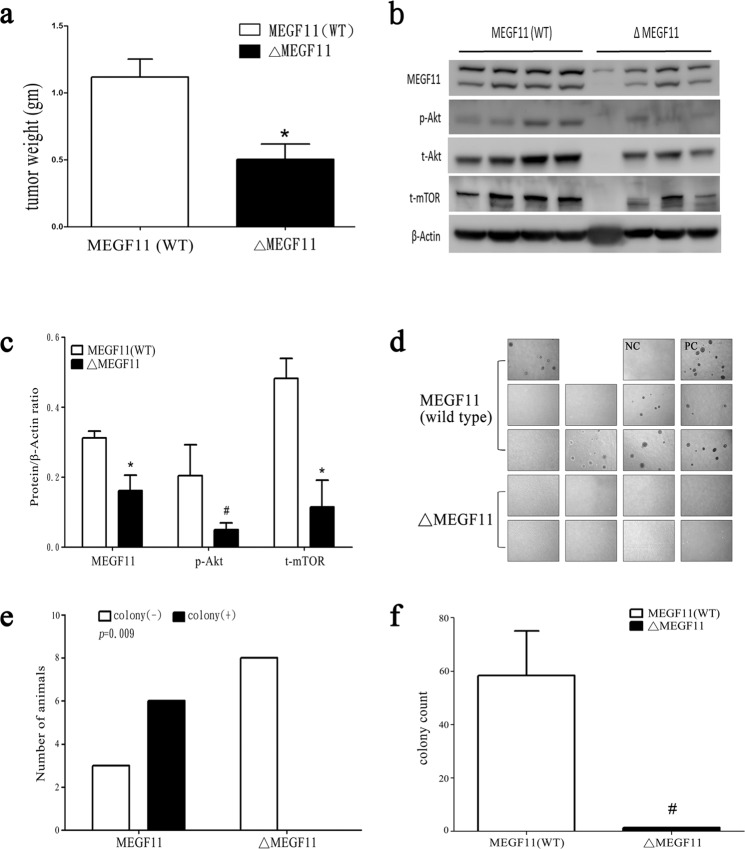
Knocked down *MEGF11* in the mouse 4T1 mammary cancer cell line decreased tumour weight and circulating tumour cells. Using the spontaneously occurring mouse mammary tumour 4T1 cell line as an *in vivo* metastatic model, 4T1 cells (1×10^7^/0.1 mL PBS) of wild type MEGF11 (n = 9) and Δ*MEGF11* 4T1 (n = 8) were orthotopically injected into two fat pads (left upper and right lower mammary glands). After two weeks, the 4T1-bearing mice were anaesthetized, and blood cells were collected and centrifuged with Ficoll-Paque (density: 1.084). The tumour weight (**a**) was measured. The implanted tumours were homogenized and analysed by Western blot (**b**,**c**). The peripheral mononuclear cells were harvested for primary cultures, and circulating 4T1 cells were selected with 6-thioguanine (60 µM) (**d**), which was analysed by Fisher’s exact test, followed by quantification by agar assay (**e**,**f**). A p value <0.05 indicates statistical significance in Δ*MEGF11* 4T1 cells compared to the wild type by Mann-Whitney U test (*) or by Student’s t test (#). NC, negative control; PC, positive control.

In addition to the above experiments involving human samples, we also used a spontaneously occurring mouse mammary tumour cell line, 4T1. We used this cell line to help us elucidate the role of MEGF11 using a mouse metastatic model. After MEGF11 was knocked down in 4T1 cells (Δ*MEGF11* 4T1) (Supplementary Information 6), there was a decrease in implanted tumour weight (Fig. 7a) and AKT-mTOR signalling (Fig. 7b,c) compared to the wild type MEGF11. By measuring the number of circulating 4T1 cells using 6-thioguanine as a selection and an agar assay, there was a significant decrease in the number of circulating Δ*MEGF11* 4T1 cells (Fig. 7d) (Fig. 7e,f) compared to the MEGF11 wild type.

## Discussion

Previous studies have suggested that MEGF11 is involved in the formation of mosaics^24^ and in haematopoietic differentiation^23^. Using cDNA open array analysis of 224 genes using paired TNBC tissue samples (16 recurrent and 24 non-recurrent tissues), we found that MEGF11 is significantly upregulated in tumour tissue samples associated with subsequent clinical recurrence compared to the tumour tissue samples without recurrence. In this study, we are the first to demonstrate that MEGF11 has a role in the mechanisms associated with breast cancer recurrence.

There is evidence that dysregulation of AKT-mTOR signalling, such as AKT overexpression, PI3K amplification/mutation, and loss of PTEN function, plays an important role in the oncogenesis of many cancers^26^, including one subtype of triple negative breast cancer^8,27^. Our results show that knockdown of *MEGF11* in TNBC cell lines is able to bring about a significant decrease *in vitro* and *in vivo* cell proliferation activity via inhibition of AKT, mTOR and NF-κB signalling. This suggests that MEGF11 is likely to be essential to the modulation of cell growth. Furthermore, no circulating mouse 4T1 cells were selected by 6-thioguanine in *the ∆MEGF11* 4T1 line, which suggests that MEGF11 also plays an important role in tumour metastasis^28^. It should be noted that our co-localization studies demonstrate that MEGF11 does not co-localize with EGFR or Gs protein in TNBC cells (Supplementary Information 7).

Because stromal cells and immune cells within and around the tumour microenvironment have been shown to play important roles in predicting the prognosis and progression of cancer in a patient, check point immunotherapy for breast cancer, using various targeted monoclonal antibodies, has begun to be used alongside conventional treatments, such as chemotherapy or endocrinal therapy^29–31^. The interaction between cancer cells within a given microenvironment involves not only cell-cell interactions but also the release of many cytokines and chemokines. For example, the presence of TNF-α^32^, IL-21^33^ and IL-17 has been demonstrated to correlate negatively with patient prognosis and with their chemoresistance to paclitaxel^34^. Recent evidence suggests that IL-17A modulates the tumour microenvironment via recruitment of immune cells, including myeloid-derived suppressor cells (MDSCs), Th17 cells and neutrophils^35,36^. Interestingly, our results show that over-expression of MEGF11 in TNBC cells does not increase cell proliferation, but rather triggers the expression of many cytokines and chemokines, which will effectively result in a cytokine cascade. Furthermore, a positive feedback between MEGF11 and IL-17A in MDA-MB-231/468 was also demonstrated in this study, which might explain the role of MEGF11 in TNBC recurrence.

Recruitment of immune cells is well known to be associated with the attenuation of anti-tumour immunity and an increase in anti-therapy effects. In addition to IL-17A, many other chemokines are involved in breast cancer progression via paracrine regulation. For example, breast cancer-derived CXCL1/2 is able to attract CD11b^+^Gr1^+^ myeloid cells, and this then promotes cell survival and metastasis^37^. Recent evidence suggests that CXCL5 promotes bone metastasis in breast cancer via the ERK/MSK1/Elk-1/Snail signalling pathway^38^. Furthermore, CCL20 increases cell proliferation and migration via the AKT and MAPK signalling pathways^39^. Our results show that upregulation of *MEGF11* does significantly increase chemokine expression, which further supports the abovementioned findings.

In addition to immune cells, the tumour microenvironment also involves endothelial cells. Our previous study demonstrated that BDNF promotes the migratory activity of both tumour cells (MDA-MB-231) and endothelial cells (HUVECs) via a number of autocrine and paracrine regulation pathways, respectively. In addition to overexpression of TrkB, a BDNF receptor, there is a significant inverse association with survival outcome among TNBC patients^40^. The present study shows that over-expressed *MEGF11* upregulates the gene expression of *BDNF/TrkB* in TNBC cells (Supplementary Information 8), which suggests that MEGF11 plays a role in tumour cell-endothelial cell interactions.

Until the present study, there has been no information available on the role of MEGF11 in breast cancer. Our results demonstrate that MEGF11 play important role in tumour survival and that overexpression of MEGF11 induces both cytokine and chemokine cascades, which in turn will bring about modulation of the tumour microenvironments within TNBC cells.

## Conclusions

We conclude that MEGF11 might be a potential therapeutic target that could be used in future TNBC treatment.

## Subjects and Methods

### Subjects

Study protocols that involved human tumour tissue from a bio-bank followed the hospital guidelines and were approved by the Institutional Review Board of Taipei Veterans General Hospital (2013-10-020BC). From Jan. 2001 to Dec. 2010, breast cancer patients who were diagnosed based on their tissue samples were identified at our hospital. One hundred thirty-five patient records, including information on oestrogen receptor (ER) status, progestin receptor (PR) status, HER2 status and clinical outcome, including overall survival (OS) and recurrence-free survival (RSF), were retrospectively reviewed from the hospital database. All data had been collected during clinical care and did not involve direct contact with the patients whose information had been collected and analysed; consequently, written consent by the study subjects was waived by the Institutional Review Board. Every subject enrolled in this study (n = 135) was followed up for ≥5 years, with the shortest and longest follow-up times being 60 months and 95 months, respectively. All patients were subjected to a Kaplan-Meier survival analysis. ER or PR values of ≥1% were defined as positive, while ER or PR values of <1% were defined as negative.

### Immunohistochemical analysis of MEGF11 expression

The protein expression levels of MEGF11 from a tissue array (135 tumour samples), obtained from the archives of the Department of Pathology, were assessed by immunohistochemical staining for MEGF11 (Genetex, GTX49102). The results were analysed by one pathologist over a short period of time (3 months). The protein expression of MEGF11 was semi-quantified and expressed as (0), <10%, (1), 11–25%, (2), 26–50%, and (3)>50% of the tumour cells examined.

### Cell line and reagents

The human triple negative breast cancer cell lines MDA-MB-231 and MDA-MB-468, which are ER low and HER2 low, respectively, were maintained in F12 MEM (NO.12400-024, Gibco, NY, USA). The mouse mammary tumour cell line 4T1 ^[26]^ was cultured in RPMI medium. These cell lines were obtained from American Type Culture Collection (ATCC, Manassas, VA, USA). The media were supplemented with 10% FBS, 2 mM L-glutamine and penicillin/streptomycin, and the cells were cultured at 37 °C in a humidified atmosphere containing 5% CO_2_. All cell lines were tested and shown to be mycoplasma-free.

### Short hairpin RNA (shRNA) transfection

Short hairpin RNA (shRNA), obtained from Academia Sinica, was used to silence the *MEGF11* gene. One day after the MDA-MB-231, MDA-MB-468 or mouse 4T1 cell lines were subcultured, the cells (30 to 40% confluence) were transfected for 24 h with either a shRNA that targeted *MEGF11* or a non-silencing control shRNA using GenePORTER 2 transfection reagent (Genlantis, San Diego, CA, USA) that had been dissolved in Optimem (Invitrogen) at a final concentration of 80 nM. Next, human MDA-MB-231 cells, human MDA-MB-468 cells or mouse 4T1 cells were recovered and used for the various experiments. After several passages, ∆*MEGF11* MDA-MB-231 and ∆*MEGF11* 468 cell lines and a ∆*MEGF11* 4T1 cell line were established by puromycin selection.

### Creation of the MEGF11 expression vector

The MEGF11 expression vector was generated by amplification of the full-length *MEGF11* cDNA from human MDA-MB-231 cells using a specific primer pair (forward primer: 5′-GCGATCGCCATGGTGCTCTCCCTGAC-3; reverse primer: 5′-ACGCGTAGATTGCTTGTCCTGGGACG-3′); this fragment was then cloned into a pCMV-AC-GFP vector (Origene #PS100010). The construct was verified by DNA sequencing. Next, lentivirus containing the *MEGF11* construct was created by Academia Sinica, ROC and used for further studies.

### Cell growth measured by trypan blue dye exclusion assay and cell cycle analysis

MDA-MB-231 and MDA-MB-468 cells were transferred into low serum culture medium at a cell density of 1 ×10^4^ per well using 12-well plates. After 1, 2, 3 and 4 days of seeding, the cells were washed twice with phosphate buffered saline (PBS), pH 7.4, and trypsinized using 0.5 mL trypsin-ethylenediamine tetraacetic acid (0.05% trypsin, 0.53 mL ethylenediamine tetraacetic acid I 4Na, Gibco/Invitrogen, New York, NY). Next, the suspended cells were re-suspended in fresh culture medium, and this was followed by counting of cell numbers using a haemocytometer-based trypan blue dye exclusion cell quantification assay system. Cell cycles were calculated as cell cycle fraction percentages, namely, sub G_0_/G_1_ phase, G_0_/G_1_ phase, S phase, and G_2_/M phase.

### Cell migration assay

*In vitro* cell migration assays of MDA-MB-231 cells (the MDA-MB-468 cell line is not suitable for this assay) were performed using a cell culture insert^41^ (NO.80209, ibidi, Munich, Germany). In brief, 2 × 10^4^ cells were seeded within an insert on a 3.5 cm Petri dish overnight, which was followed by low serum (1% FBS) starvation for 24 h. Next, the cells were washed with PBS, the inserts were removed, and the cells were cultured for another 24 h. During this period, cell migration took place and was observed; this involved the cells being examined under a light microscope and photographed. The percentage migration of the cells was calculated compared to a control.

### Western blot analysis

Cultured cells were lysed in a buffer containing 150 mM KCl, 10 mM Tris pH 7.4, 1% Triton X-100, phosphatase inhibitor and protease inhibitors cocktail (Complete Mini; Roche, Mannheim, Germany). The protein concentration of each cell homogenate was then measured using Bradford’s method^42^. Next, 30 μgm of protein was loaded and separated using 10% SDS-PAGE; the proteins were then transferred to a nitrocellulose membrane (Hybond-C; Amersham Biosciences, NJ, USA). Each membrane was blocked using 5% bovine serum albumin and, finally, the proteins were probed using specific primary antibodies (Supplementary Information 9) that had been obtained commercially. The specificity of anti-MEGF11 antibody (Genetex, GTX49102) was validated by cell lines and human tissues (Supplementary Information 10). The unprocessed blotted images were also provided in supplementary ref. ^6^. The absence of some images of adequate length were due to unrestored damage of the image files.

### Total RNA extraction and reverse transcription PCR

Total RNA was isolated by the modified single-step guanidinium thiocyanate method^43^ (TRI REAGENT, T-9424, Sigma Chem. Co., St. Louis, MO, USA). Complementary DNA (cDNA) was prepared from the total RNA using a First Strand cDNA Synthesis Kit (Invitrogen, CA, USA). The *de novo* gene synthesis changes found for each treatment group were detected by reverse transcriptase-polymerase chain reaction (RT-PCR). Commercially available primers pairs (e.g., for MEGF11: Forward 5′-TGG CTG ACA CTT TCG AAC AC-3′; Reverse5′-CCT CAT GGA CAT GTT TGC AG-3′) were used. Possible contamination of any PCR component was excluded by performing a PCR with these components in the absence of the RT product for each set of experiments (the non-template control, NTC). For statistical comparison, the relative expression levels of specific genes at the mRNA level were normalized against the amount of *GAPD* mRNA present in the same RNA extract. All samples were analysed in triplicate.

### Ingenuity pathways analysis (IPA)

IPA is an all-in-one web-based platform that has been adopted for the analysis, integration, and interpretation of data derived from ‘omics experiments. We did not perform all transcripts, except those selected by microarray data for IPA, when MEGF-11 was overexpressed.

### *In vivo* tumour xenografting

Study protocols that involved experimental mice followed the ARRIVE (Animal Research: Reporting of *In Vivo* Experiments) guidelines and were approved by the Institutional Animal Committee of Yang-Ming University (No. 1050802) and Taipei Veterans General Hospital (No. 2018-029). Immunodeficient NU-Foxn1nu mice were obtained from the National Laboratory Animal Center (Taipei, Taiwan, ROC). They were given *ad libitum* access to food and water and maintained in a specific pathogen-free environment with a 12 h light-dark cycle at 22–24 °C and 50% humidity. The mice were used for the experiments at 8 weeks of age. Wild type and knocked down *MEGF11* (∆*MEGF11*) MDA-MB-231 cells containing a luciferase gene were injected into back of immunodeficient NU-Foxn1nu mice at a cell density of 1 × 10^7^ in 0.1 mL PBS for each mouse; this gave rise to a solid tumour that was noticeable around the injection site after between days 7 and 14. From that point, the progression of the tumour in terms of size was visualized using an *in vivo* imaging system (IVIS). For the tumour metastasis study, wild type and knocked down *MEGF11* (∆*MEGF11*) mouse mammary 4T1 cells (1 × 10^7^ in 0.1 mL PBS) were orthotopically injected into two fat pads (left upper and right lower mammary glands) of 8-wk-old female BALB/c mice. The mice were sacrificed at 8 weeks thereafter or when the tumour sizes made up more than 2% of the body weight. The tumour sizes and weights were measured, and tumour tissue or any organs suspected to be metastatic, such as the lung and liver, were frozen for further analysis.

### Selection of circulating mouse mammary breast cancer 4T1 cells

After the 4T1 bearing mice were anaesthetized, blood cells were collected and centrifuged (400 g) in Ficoll-Paque PREMIUM (density: 1.084) (17-5446-02, GE Healthcare Bio-Sciences, Sweden) gradient medium. The peripheral mononuclear cells were subjected to primary culture for several passages, and then circulating 4T1 cells were selected using 6-thioguanine (60 μM) (A4882, Sigma-Aldrich, MO, USA) ^[26]^, which was followed by quantification with a 2-hydroxyethyl agarose colony assay (A4018, Sigma-Aldrich, MO, USA). A colony was defined as a blue dye-stained group of cells that was ≥1 mm.

### Statistics

Data are presented as the means ± SD or SEM. Differences between groups were identified by one-way ANOVA and Dunnett’s *post hoc* test. Statistical comparisons between two independent groups were determined by Student’s t test or the Mann-Whitney U test. The contingency table for the presence of circulating 4T1 cells was analysed by Fisher’s exact test. A *p* value of <0.05 was considered statistically significant (GraphPad Prism 5).

RFS was defined as the time between initial breast cancer diagnosis and the date of recurrence as confirmed by pathology or an imaging study. OS was calculated from the time of initial breast cancer diagnosis to the date of death or last consultation. The Kaplan–Meier method was used to estimate the cumulative incidence of RFS and OS, and log-rank tests were then used for the various comparisons (GraphPad Prism 5).

## Declarations

### Ethics approval and consent to participate

Study protocols involving experimental mice followed ARRIVE (Animal Research: Reporting of *In Vivo* Experiments) guidelines and were approved by the Institutional Animal Committee of Yang-Ming University (No. 1050802) and Taipei Veterans General Hospital (No. 2018-029).

The human study for tumour tissue utilization from the bio-bank was approved by the Institutional Review Board of Taipei Veterans General Hospital (# 2013-10-020BC).

## Supplementary information

Supplementary information.

## Data Availability

All data generated or analysed during this study are included in this published article (and its Supplementary Information files)
