# Supplementary material for: MEGF11 is related to tumour recurrence in triple negative breast cancer via chemokine upregulation
Source: Sci Rep. 2020 May 15;10:8060. doi: 10.1038/s41598-020-64950-0 (PMC7229019; doi:10.1038/s41598-020-64950-0)

Supplementary references

MEGF11 is related to tumor recurrence in triple negative breast cancer via chemokine up-regulation

Jen-Hwey Chiu, MD, PhD[1,2,3*], Ling-Ming Tseng, MD[1,4], Tzu-Ting Huang PhD[1,5], Chun-Yu Liu, MD, PhD[1,6], Jir-You Wang, PhD[7], Ching-Po Huang[8], Yi-Fang Tsai, MD[1,9], Chih-Yi Hsu, MD[10,11]

Supplementary information 1.

In our previous study (PMID: 26458489), many genes including *MEGF11* gene was identified by differentially displayed microarray analysis in 15 paired recurrent and non-recurrent TNBC samples. Then we performed real-time PCR–based solution for high-throughput 224 gene expression analysis inpaired TNBC tissue samples (16 recurrent and 24 non-recurrent tissues), and found that MEGF11 was the top one gene that had higher transcription expression in recurrent TNBC tumors that non-recurrent ones.Each gene was analyzed in triplicate.

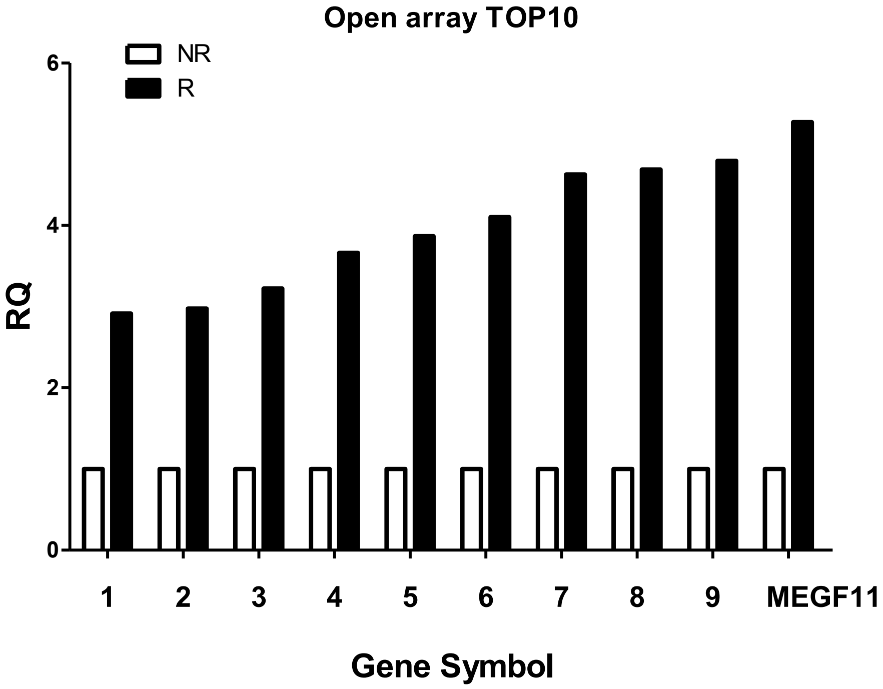

Supplementary information2.

Kaplan-Meier plotter database spit patient by upper quartile showed a negative correlation between *MEGF11* gene up-regulation and patients' DFS.The desired Affymetrix IDs is valid: 1569879_a_at (MEGF11).

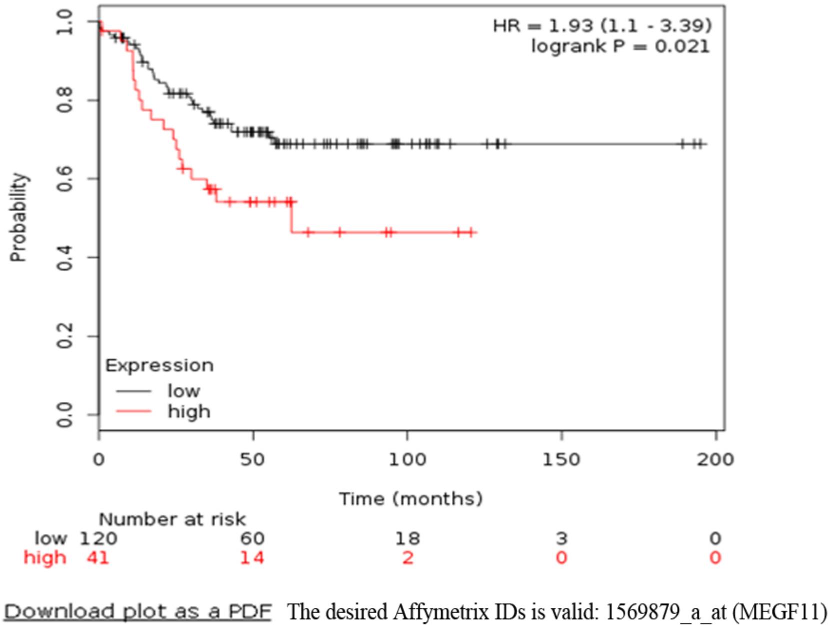

Supplementary information3.

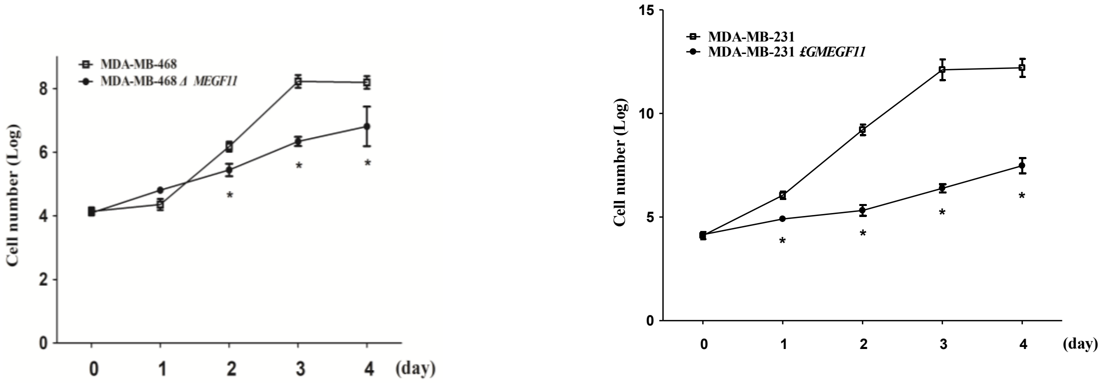

Growth curves of TNBC MDA-MB-231 and MDA-MB-468 without (wild type) and with MEGF11 knocked down (ΔMEGF11). *, p<0.05, Two-way ANOVA.

Supplementary information4.

Cell cycle analysis in o/e MEGF11 MDA-MB-231/468 cells disclosed no significant change in S- or S+G2M phase fraction compared to the scramble group.

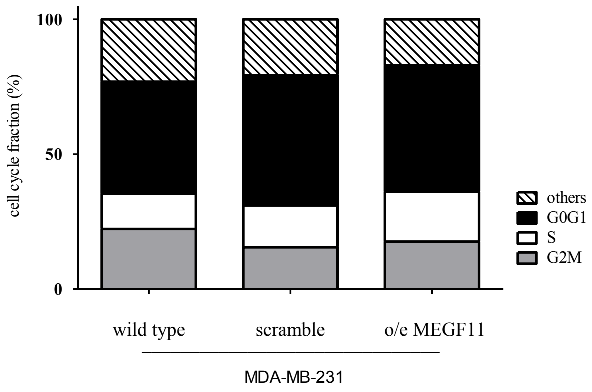

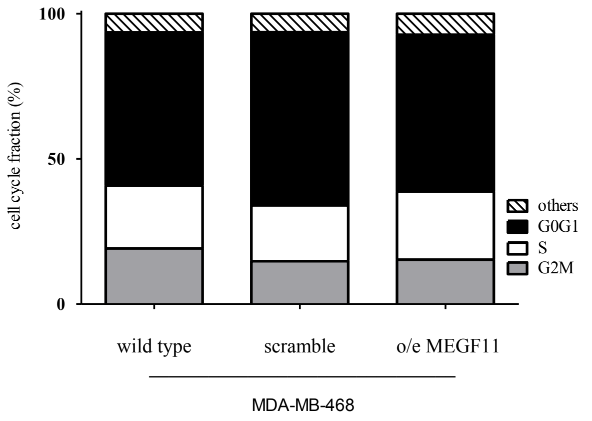

Supplementary information5.

Ingenuity Pathway Analysis (IPA) for MEGF11-related gene expression.

Top Canonical Pathways

Name	p-value	Overlap	
Granulocyte Adhesion and Diapedesis	8.69E-11	3.9%	7/179
Agranulocyte Adhesion and Diapedesis	1.32E-10	3.7%	7/190
Role of IL-17A in Psoriasis	2.65E-10	30.8%	4/13
Role of IL-17A in Arthritis	3.10E-07	5.8%	4/69
Hepatic Fibrosis/ Hepatic Stellate Cell Activation	4.10E-07	2.7%	5/187

Top Disease and Disorders

Name	p-value	#Molecules
Inflammatory Response	8.50E-04-2.92E-14	15
Hematological Disease	8.50E-04-1.57E-09	7
Immunological Disease	8.50E-04-1.57E-09	11
Organismal Injury and Abnormalities	8.50E-04-1.57E-09	16
Gastrointestinal Disease	8.50E-04-1.47E-08	13

Categories	p-Value	Molecules	# Molecules
Cell-To-Cell Signaling and Interaction	5.62E-15	CCL20,CD14,CXCL1,CXCL2,CXCL3,CXCL8,GDF15,I	10
Cell-To-Cell Signaling and Interaction	9.42E-15	CCL20,CD14,CX3CL1,CXCL1,CXCL2,CXCL3,CXCL8	11
Cell-To-Cell Signaling and Interaction	2.92E-14	CCL20,CD14,CX3CL1,CXCL1,CXCL2,CXCL3,CXCL8	10
Cell-To-Cell Signaling and Interaction	1E-13	CCL20,CD14,CXCL1,CXCL2,CXCL3,CXCL8,IL1R1,IL	9
Cellular Movement, Hematological Sy	1.88E-13	CCL20,CD14,CX3CL1,CXCL1,CXCL2,CXCL3,CXCL8	12
Cellular Movement, Hematological Sy	4.12E-13	CCL20,CD14,CX3CL1,CXCL1,CXCL2,CXCL3,CXCL8	13
Cell-To-Cell Signaling and Interaction	1.91E-12	CCL20,CD14,CX3CL1,CXCL1,CXCL2,CXCL3,CXCL8	10
Cellular Movement, Hematological Sy	4.07E-12	CD14,CX3CL1,CXCL1,CXCL2,CXCL3,CXCL8,GDF15	10
Cellular Movement, Hematological Sy	8.26E-12	CCL20,CD14,CX3CL1,CXCL1,CXCL2,CXCL3,CXCL8	11
Cell-To-Cell Signaling and Interaction	1.01E-11	CXCL1,CXCL2,CXCL3,CXCL8,GDF15,IL1R1,IL6	7
Cell Death and Survival	1.34E-11	CD14,CX3CL1,CXCL1,CXCL2,CXCL8,IL6,LCN2,NAD	8
Cell Death and Survival	1.91E-11	CD14,CX3CL1,CXCL1,CXCL2,CXCL8,IL6,LCN2,NAD	8
Cellular Movement, Hematological Sy	1.96E-11	CD14,CX3CL1,CXCL1,CXCL2,CXCL3,CXCL8,IL1R1,	9
Cell Death and Survival	2.36E-11	CXCL1,CXCL2,CXCL8,IL6,LCN2,NAD+	6
Cellular Movement, Skeletal and Mus	2.6E-11	CXCL1,CXCL2,CXCL3,CXCL8	4
Hematological System Development a	5.67E-11	CXCL1,CXCL2,CXCL3,CXCL8,IL1R1,LCN2	6
Hematological System Development a	6.3E-11	CX3CL1,CXCL1,CXCL2,CXCL3,CXCL8,IL1R1,LCN2	7
Cellular Movement, Hematological Sy	7.51E-11	CCL20,CX3CL1,CXCL1,CXCL2,CXCL3,CXCL8,IL1R1	9
Cell-To-Cell Signaling and Interaction	9.77E-11	CX3CL1,CXCL1,CXCL2,CXCL3,CXCL8,IL1R1,IL6	7
Cell-To-Cell Signaling and Interaction	1.78E-10	CXCL1,CXCL2,CXCL3,CXCL8,IL1R1,IL6	6
Cellular Movement	1.89E-10	CX3CL1,CXCL2,CXCL3,CXCL8,GDF15,IL6,STC1	7
Hematological System Development a	2.06E-10	CX3CL1,CXCL1,CXCL2,CXCL3,CXCL8,IL1R1,IL6,LC	8
Cellular Movement, Hematological Sy	2.18E-10	CX3CL1,CXCL1,CXCL2,CXCL3,CXCL8,IL1R1,LCN2	7
Cell-To-Cell Signaling and Interaction	2.53E-10	CCL20,CX3CL1,CXCL3,CXCL8,IL1R1,IL6	6
Cell-To-Cell Signaling and Interaction	2.84E-10	CD14,CXCL1,CXCL3,CXCL8,IL6,LCN2	6
Cellular Movement, Hematological Sy	2.9E-10	CD14,CX3CL1,CXCL2,CXCL3,CXCL8,IL1R1,IL6,LCN	9
Inflammatory Response	3.46E-10	CCL20,CD14,CX3CL1,CXCL1,CXCL2,CXCL3,CXCL8	11

Supplementary information 6.

For the tumor metastasis study, wild type, and knocked down MEGF11 (Δ*MEGF11*) mouse mammary 4T1 cells were orthotopically injected into two fat pads (left upper and right lower mammary glands) of 8-wk female BALB/c mice. We used two clones of Δ*megf11* lentivirus, 119472 and 119473, to knock down MEGF11 expression, followed by validation by Western blot analysis. The results demonstrated that the clone of 119472 had better efficiencyto knock down MEGF11 expression than the 119473 clone (A, B).

A

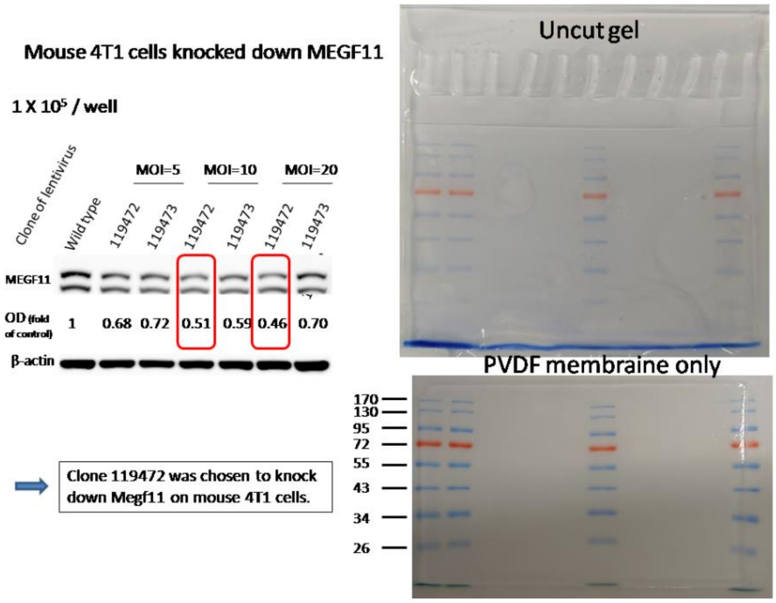

B

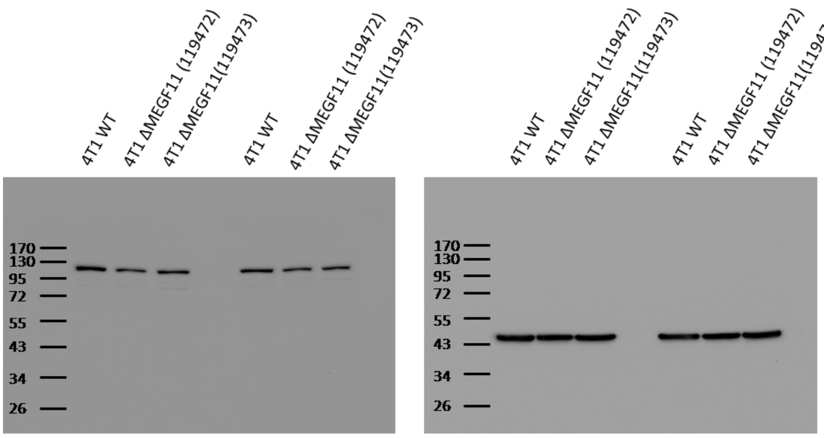

PVDF membrane hybridized with anti-MEGF11 antibody (GTX 49102) (1: 1000)

PVDF membrane hybridized with anti-beta actin antibody (1: 1000)

After the animals were sacrificed, the implanted tumors removed from wild type or ΔMEGF11 mice were analyzed with Western blot (C) and immunohistochemistry (D). The results showing that there were decreased MEGF11 expression in the tumors of ΔMEGF11 mice compared to the wild type, both in Western blot and immunochemistry analysis.

C

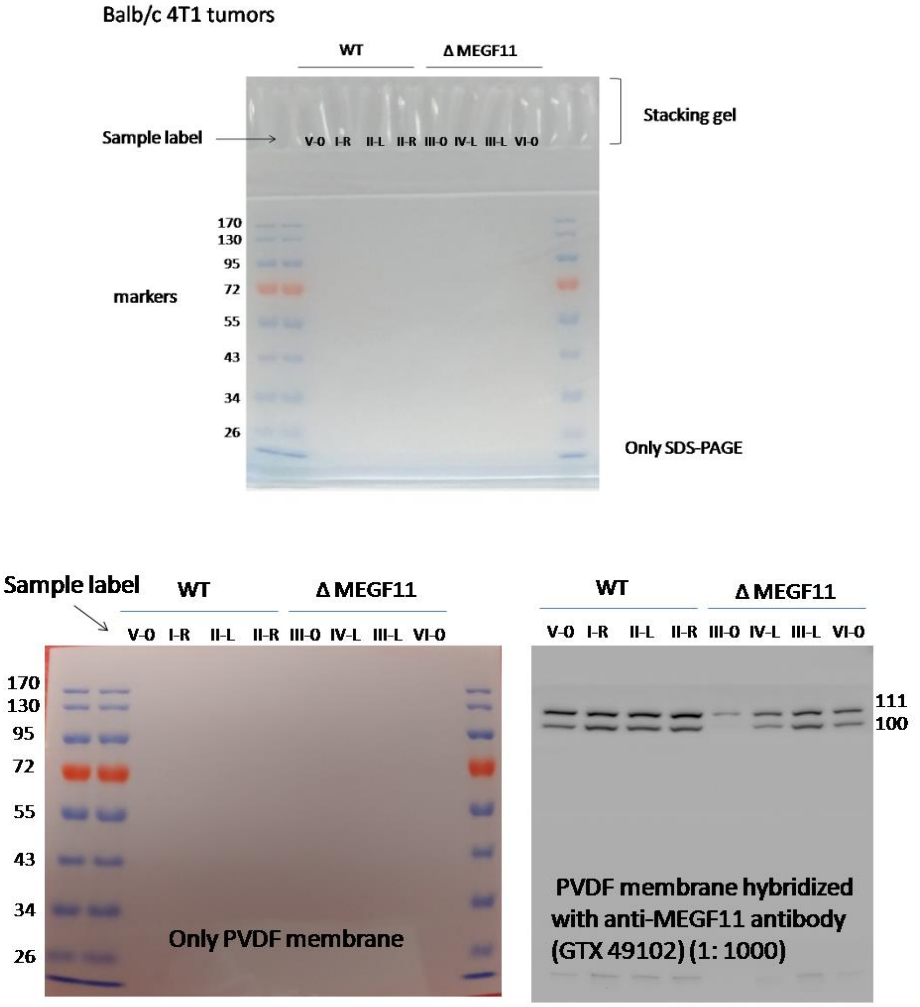

D

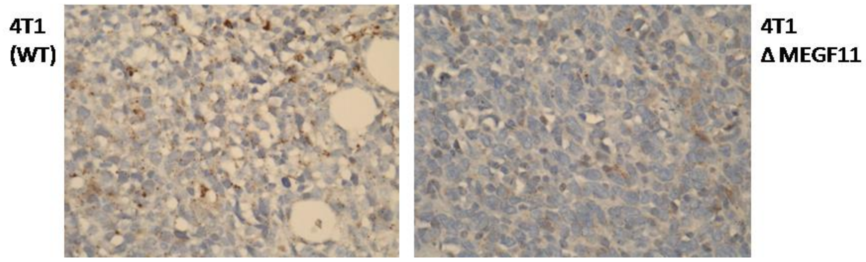

The unprocessed blots for Western blot in this manuscript are demonstrated as the followings.

Fig. 2A

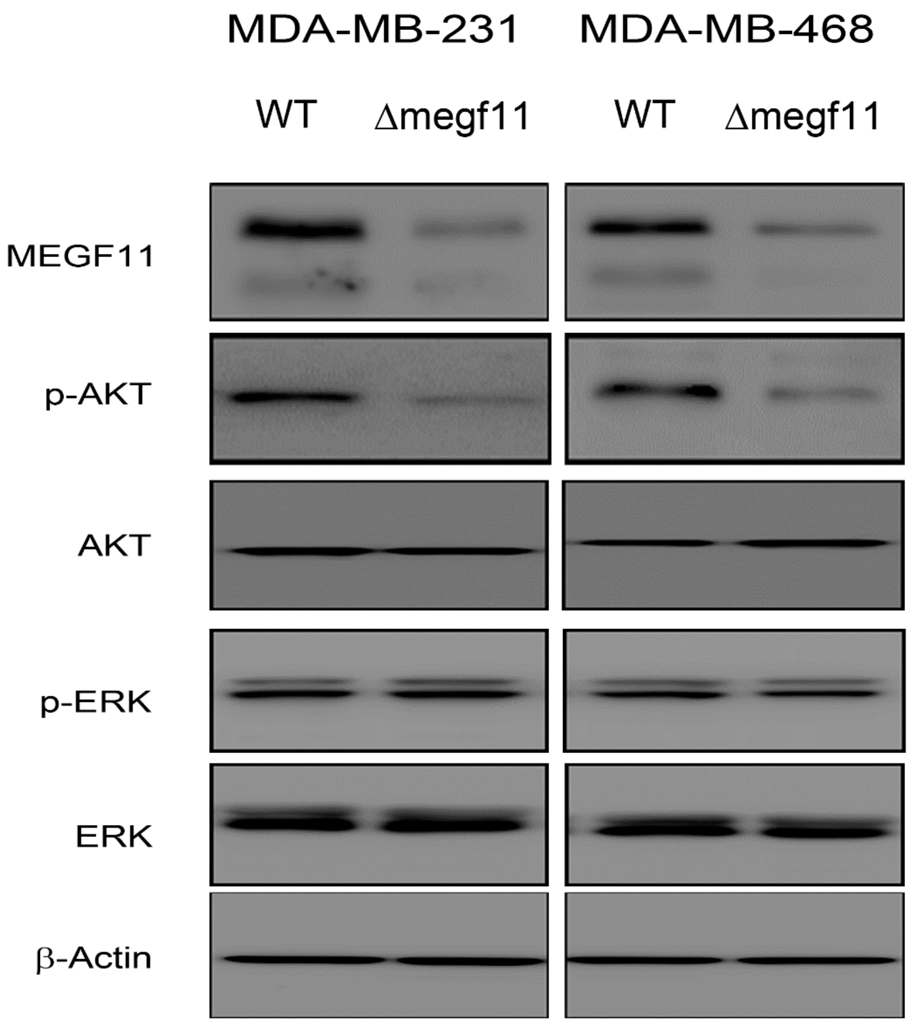

Fig. 2B

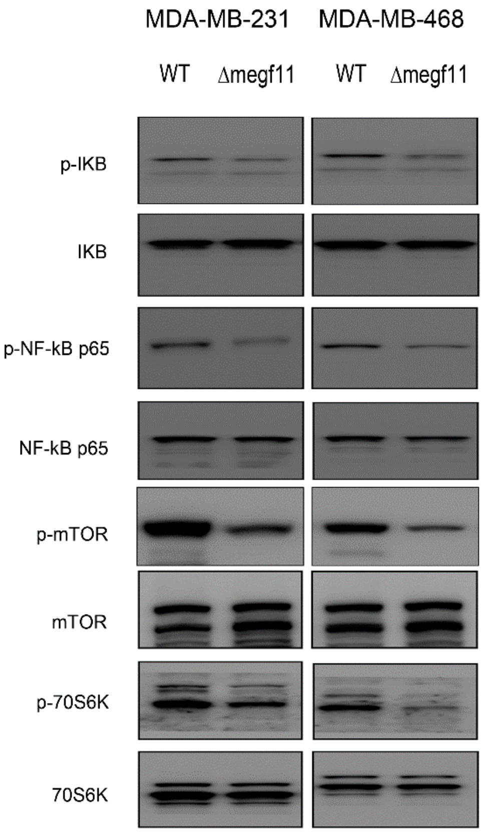

Fig. 2C

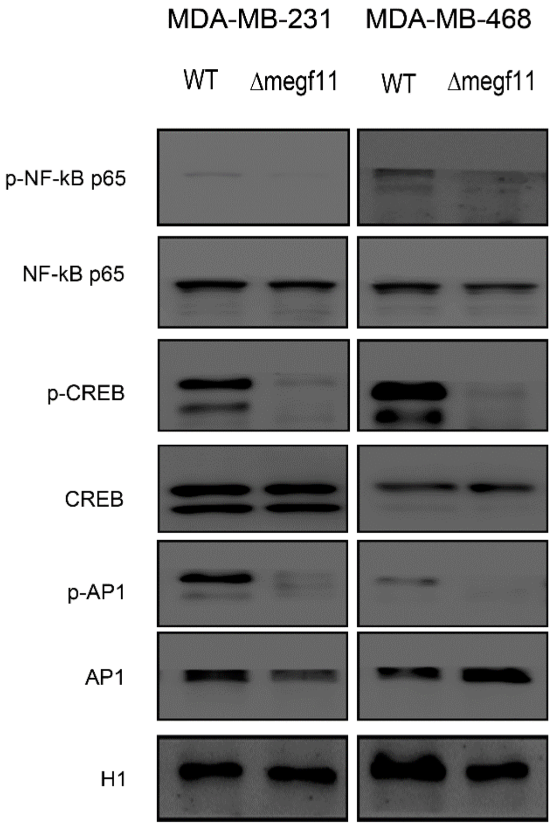

Fig. 3C

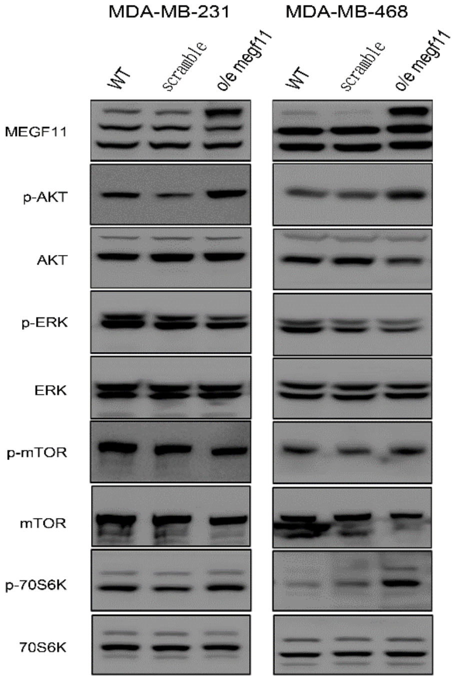

Fig. 3E

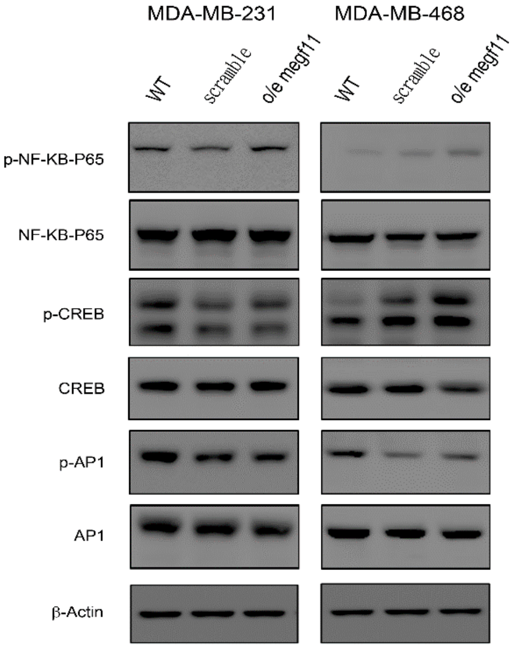

Fig. 4B

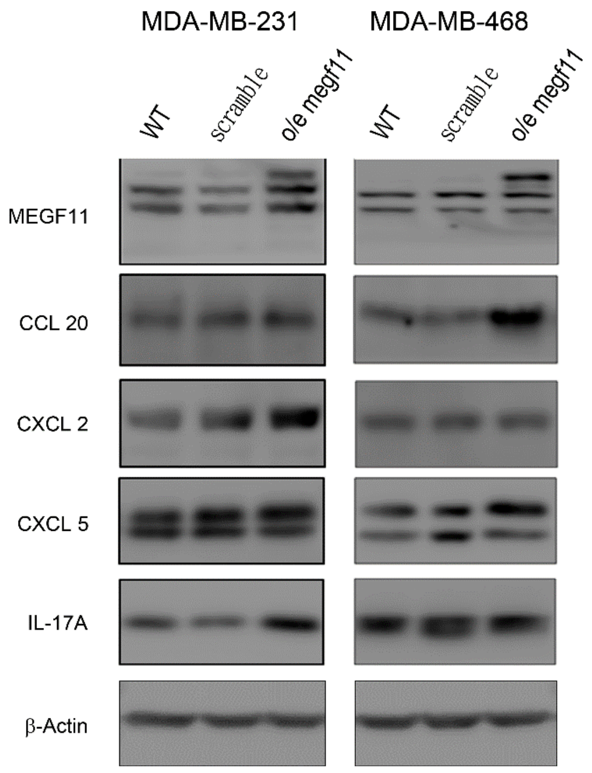
MDA-MB-231
MDA-MB-468
WT
scramble
o/e megf11
WT
scramble
o/e megf11
MEGF11
CCL 20
CXCL 2
CXCL 5
IL-17A
β-Actin

Fig. 5C

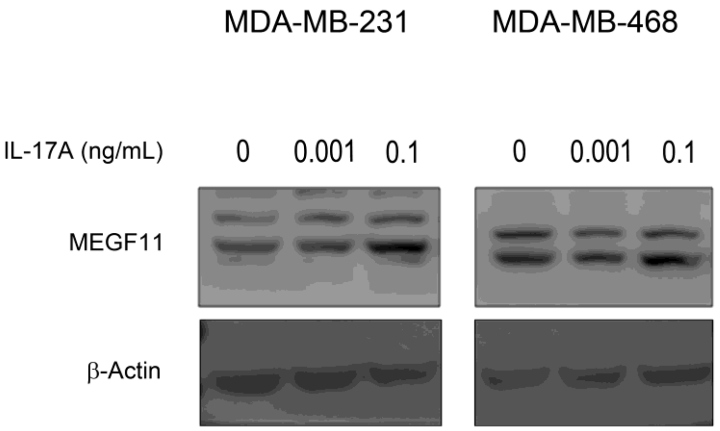
MDA-MB-231
MDA-MB-468
IL-17A (ng/mL)
0
0.001
0.1
0
0.001
0.1
MEGF11
β-Actin

Fig. 5D

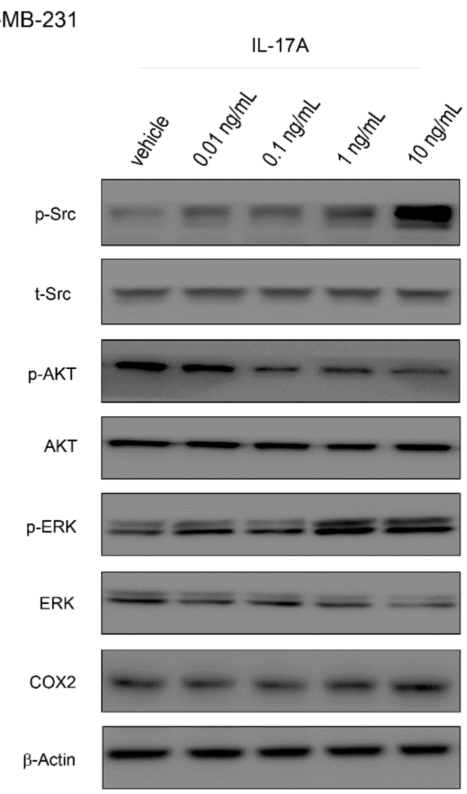

Fig. 5E

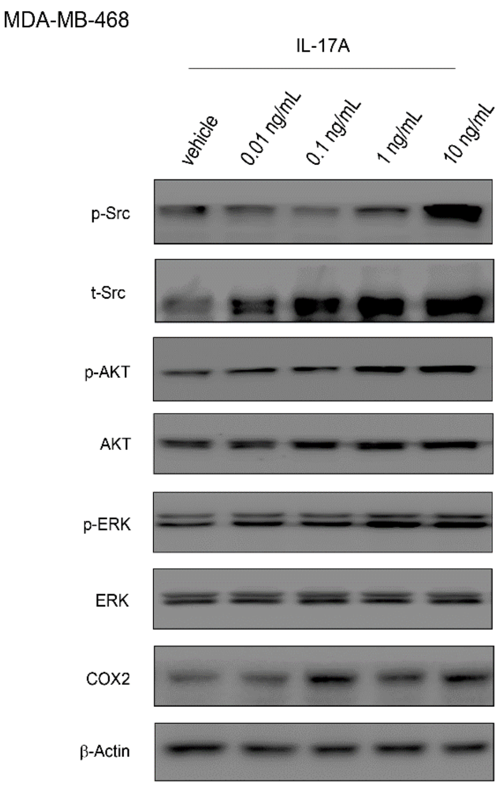

Fig. 6A

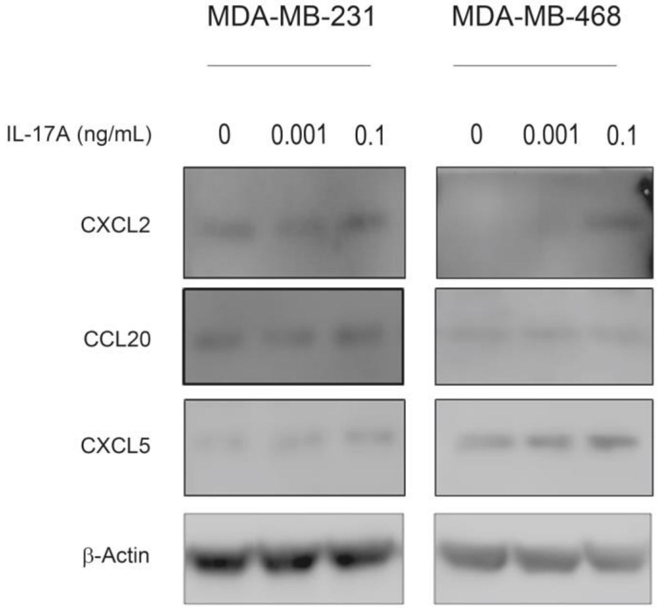

Fig. 7B

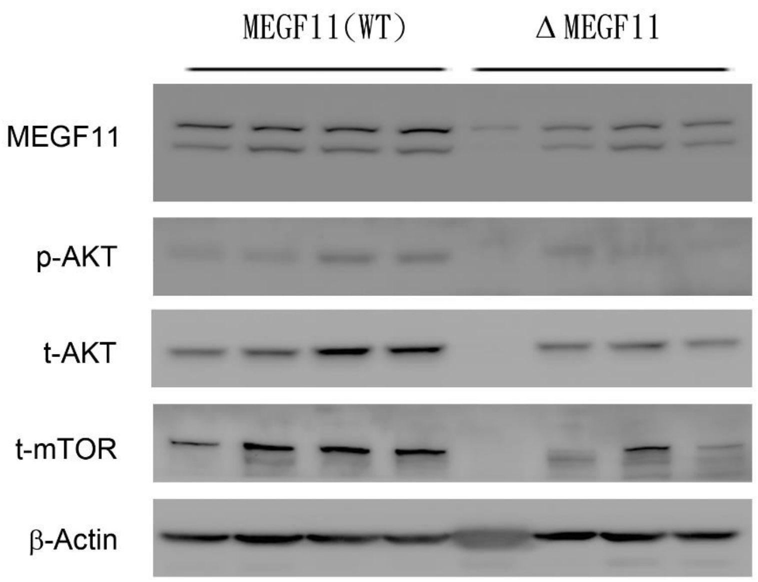

Supplementary information7.

Co-localization with immunofluorecent staining on MDA-MB-231 and MDA-MB-468. MEGF11 was localized with primary antibodies, followed by 2nd antibody conjugated with FITC (for MEGF11) or Rodamine (for Gs protein or EGFR), respectively. The results disclosed that MEGF11 protein was not co-localized with Gs protein or with EGFR in TNBC cells.

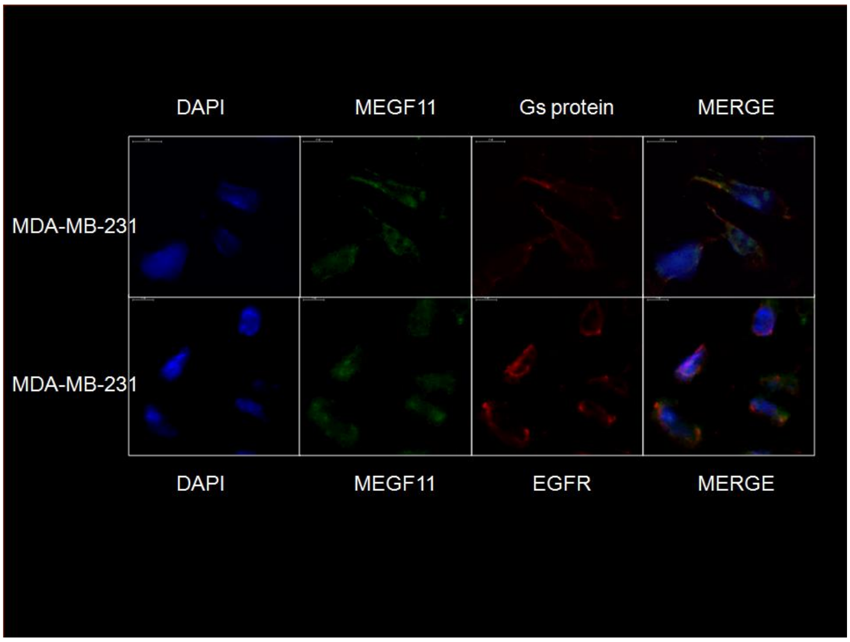

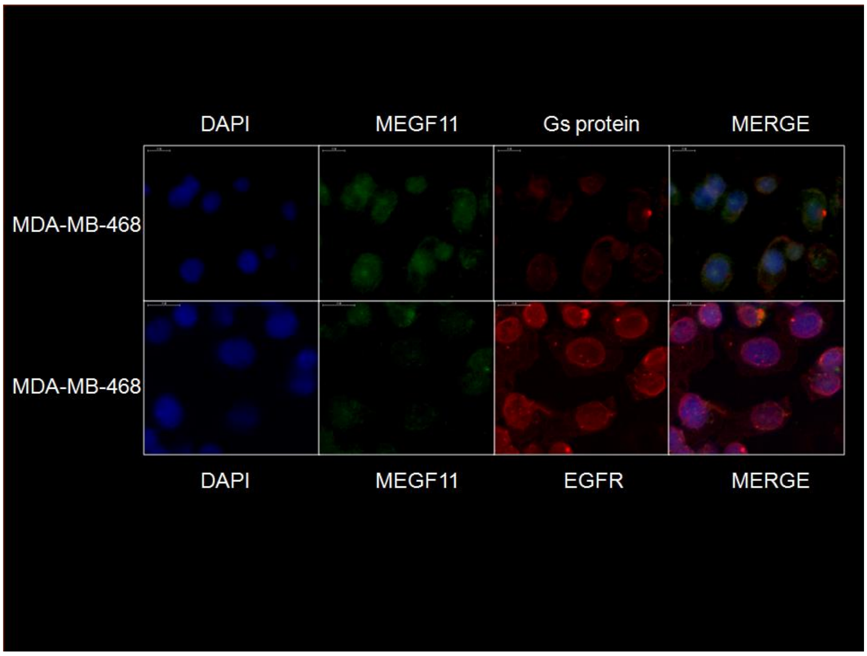

Supplementary information8.

The role of MEGF11 on BDNF and TrkB gene expression. There were up-regulated brain-derived neurotrophic factor (BDNF) and its receptor (TrkB) gene expression in TNBC MDA-MB-231 and MDA-MB-468 lines.

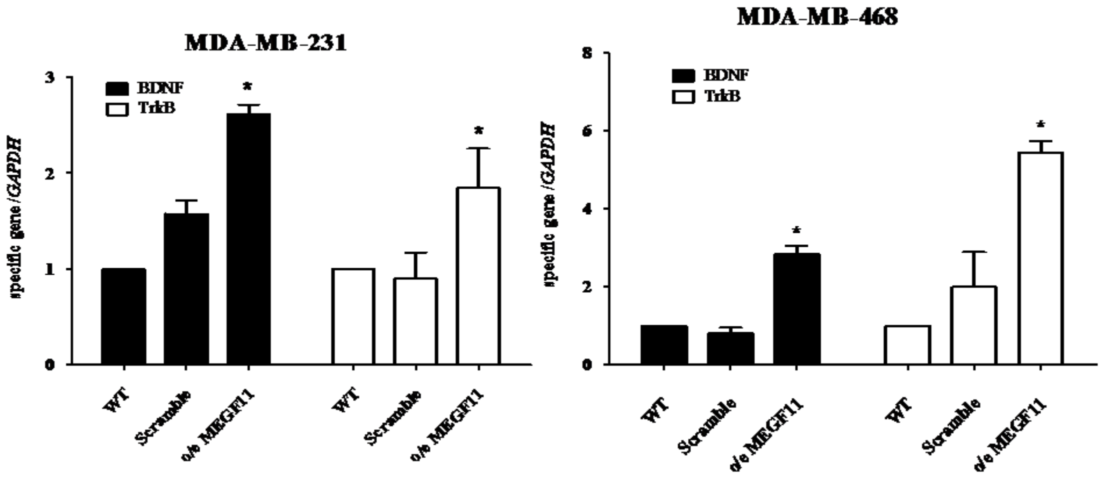

Supplementary information 9.

Antibody	Host	Clonality	Molecular (kDa)	company	catalog No.	WB titer
MEGF11	Rabbit	Polyclonal	111 (100)	Genetex	GTX49102	1:1000
p-Akt (Ser473)	Rabbit	Polyclonal	60	Cell Signaling	#9271	1:1000
p-ERK (Thr202/Tyr204)	Rabbit	Polyclonal	42, 44	Cell Signaling	#9101	1:1000
Akt	Rabbit	Polyclonal	60	Cell Signaling	#9272	1:1000
ERK [p44/42 MAPK (Erk1/2)]	Rabbit	Monoclonal	42, 44	Cell Signaling	#4695	1:1000
p-p65 (Ser536)	Rabbit	Monoclonal	65	Cell Signaling	#3033	1:1000
p65	Rabbit	Monoclonal	65	Cell Signaling	#8242	1:1000
p-CREB (Ser133)	Mouse	Monoclonal	46	Cell Signaling	#9196	1:1000
CREB	Rabbit	Monoclonal	43	Cell Signaling	#9197	1:1000
p-p70S6K (Thr389)	Rabbit	Polyclonal	70, 85	Cell Signaling	#9205	1:1000
p70S6K	Rabbit	Polyclonal	70, 85	Cell Signaling	#9202	1:1000
p-c-Jun (Ser63) (Ap1)	Rabbit	Polyclonal	48	Cell Signaling	#9261	1:1000
c-Jun (Ap1)	Rabbit	Monoclonal	43, 48	Cell Signaling	#9165	1:1000
p-mTOR (Ser2448)	Rabbit	Polyclonal	289	Cell Signaling	#2971	1:1000
m-TOR	Rabbit	Monoclonal	289	Cell Signaling	#2983	1:1000
IL-17A (for human)	Mouse	Monoclonal	17	R & D	MAB3171	1:250
beta-actin	Mouse	Monoclonal	42	Cell Signaling	#3700S	1:1000

Supplementary information 10.

To compare the specificity of different anti-MEGF11 antibody on market, we performed Western blot on MEGF11-overexpressed TNBC with two different antibodies. The results demonstrated that the antibody from Genetex (GTX49102), which was used in our studies, recognized MEGF11 protein isoforms more specifically than that from Thermo Fisher Scientific (Invitrogen, PA5-40109) (A).

A

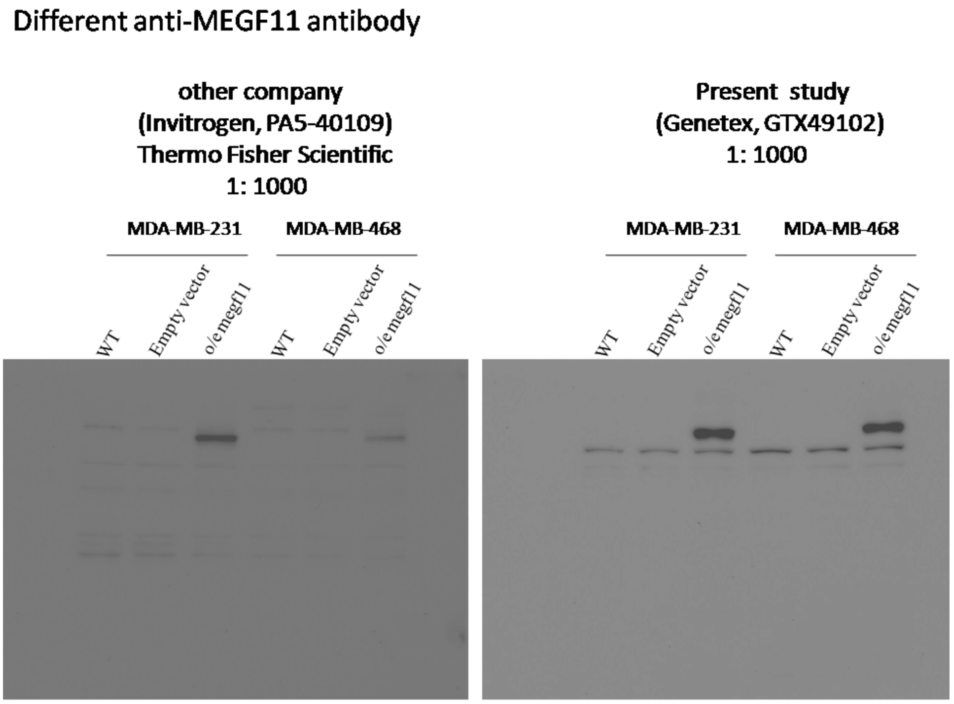

MEGF11 antibody may recognize two isoforms. Uniport shows that isoform 1 is 111 kDa and isoform 2 is 100 kDa. Our results for validation of anti-MEGF antibody (Genetex, GTX49102) demonstrated that this antibody recognizes one major (111 kDa) isoform and one minor (100 kDa) isoform of endogenous MEGF11 on wild type and Δ megf11 TNBC cells lines. Immunohistochemistry on paraffin-embedded blocks from different lines (wild type, Δmegf11, empty vector (scramble), and over-expressed *megf11* TNBC MDA-MB-231/MDA-MB-468), which were stained withanti-MEGF antibody (Genetex, GTX49102), showed that there was a decreased MEGF11 expression in Δ*megf11* lines than wild type (B).

B

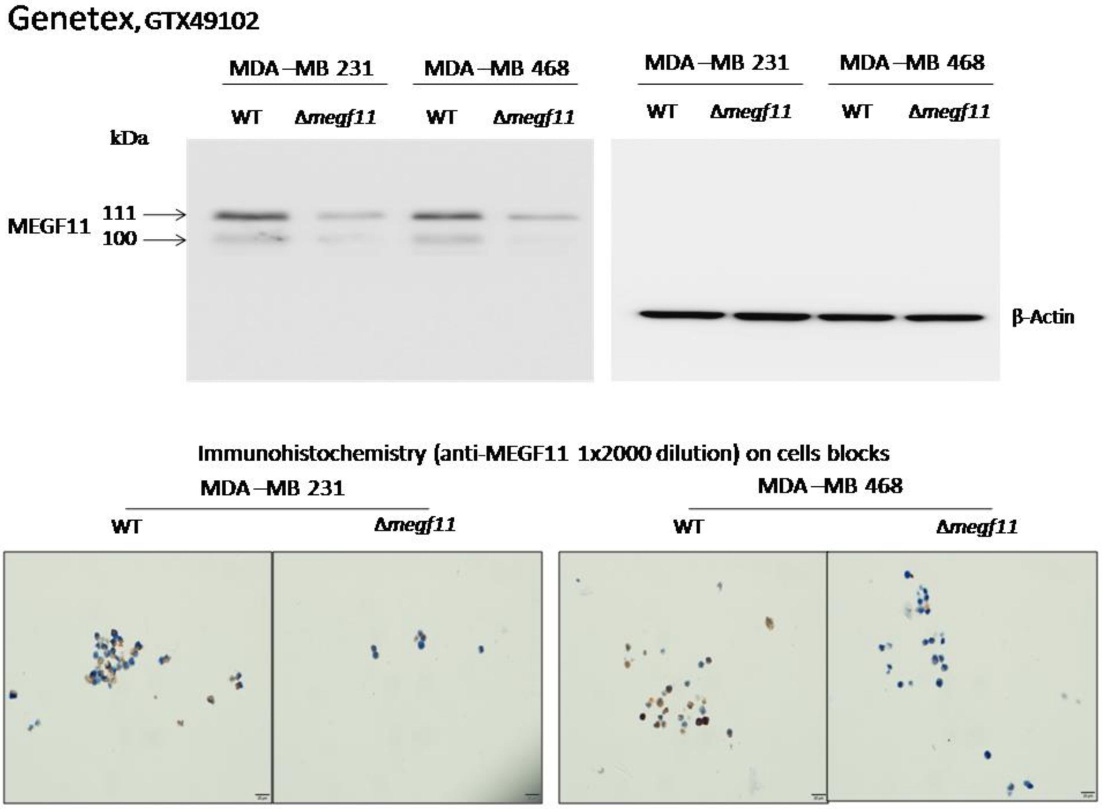

We constructed the MEGF11 over-expression vector that was generated by amplification of the full-length MEGF11 cDNA and cloned into a GPF-containing vector. Therefore, the anti-MEGF11 antibody recognized a third (137 kDa) MEGF11-GFP fusion protein, in addition to two endogenous MEGF11 isoforms (111 kDa and 100 kDa), on MEGF11 over-expressed TNBC cell lines (C). Immunohistochemistry staining also showed that there was an increased MEGF11 expression in o/e *megf11* lines than wild type (D).

..

C

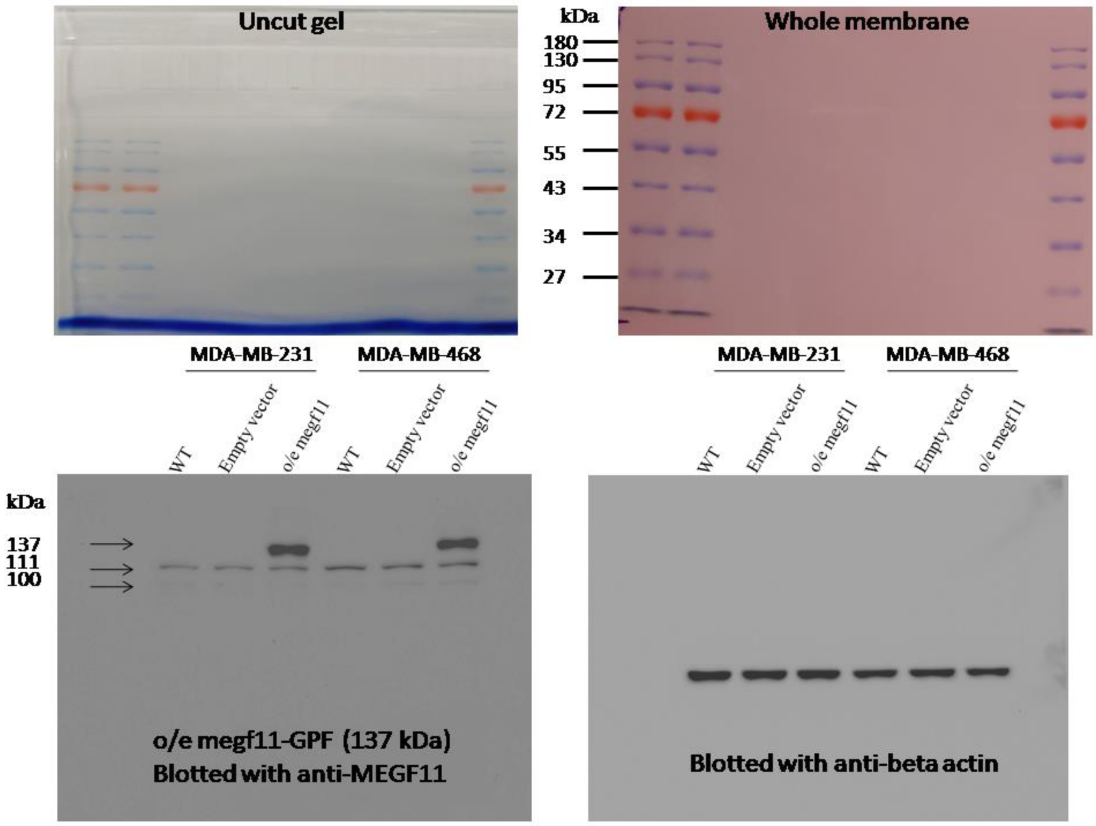
Uncut gel
Whole membrane
kDa
180
130
95
72
55
43
34
27
MDA-MB-231
MDA-MB-468
WT
Empty vector
o/e megf11
WT
Empty vector
o/e megf11
MDA-MB-231
MDA-MB-468
WT
Empty vector
o/e megf11
WT
Empty vector
o/e megf11
kDa
137
111
100
o/e megf11-GPF (137 kDa)
Blotted with anti-MEGF11
Blotted with anti-beta actin

D

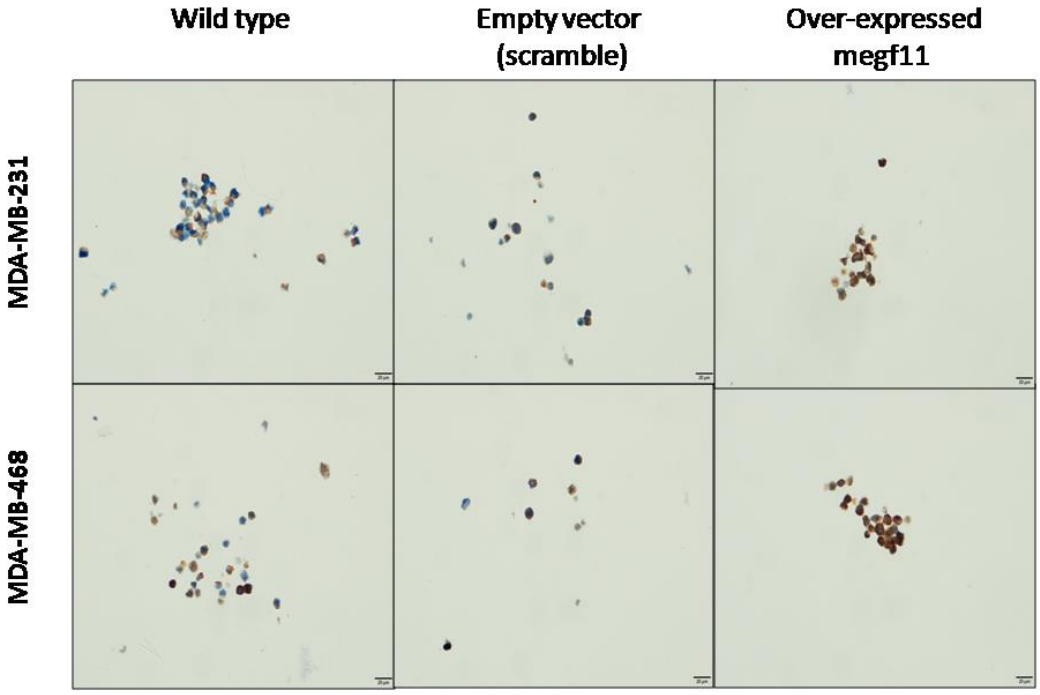
Wild type
Empty vector
(scramble)
Over-expressed
megf11
MDA-MB-231
MDA-MB-468

According to “The Human Protein Atalas”, MEGF11 is classified as predicted intracellular and membrane protein. Our IHC studies demonstrate that this antibody recognizes intracellular MEGF11 protein. Human skin tissue is used as a MEGF11(+) control, while uterine cervix, prostate and pancreas tissues are used as MEGF11 (-) control. Our results disclose that no back ground (non-specific) staining on these MEGF11(-) tissues but a specific staining on MEGF11(+) tissue (skin) are noticed, indicating the specificity of this antibody. Besides, the MEGF11 expression on different TNBC tissue ranges from (-) to (+++) (E).

E

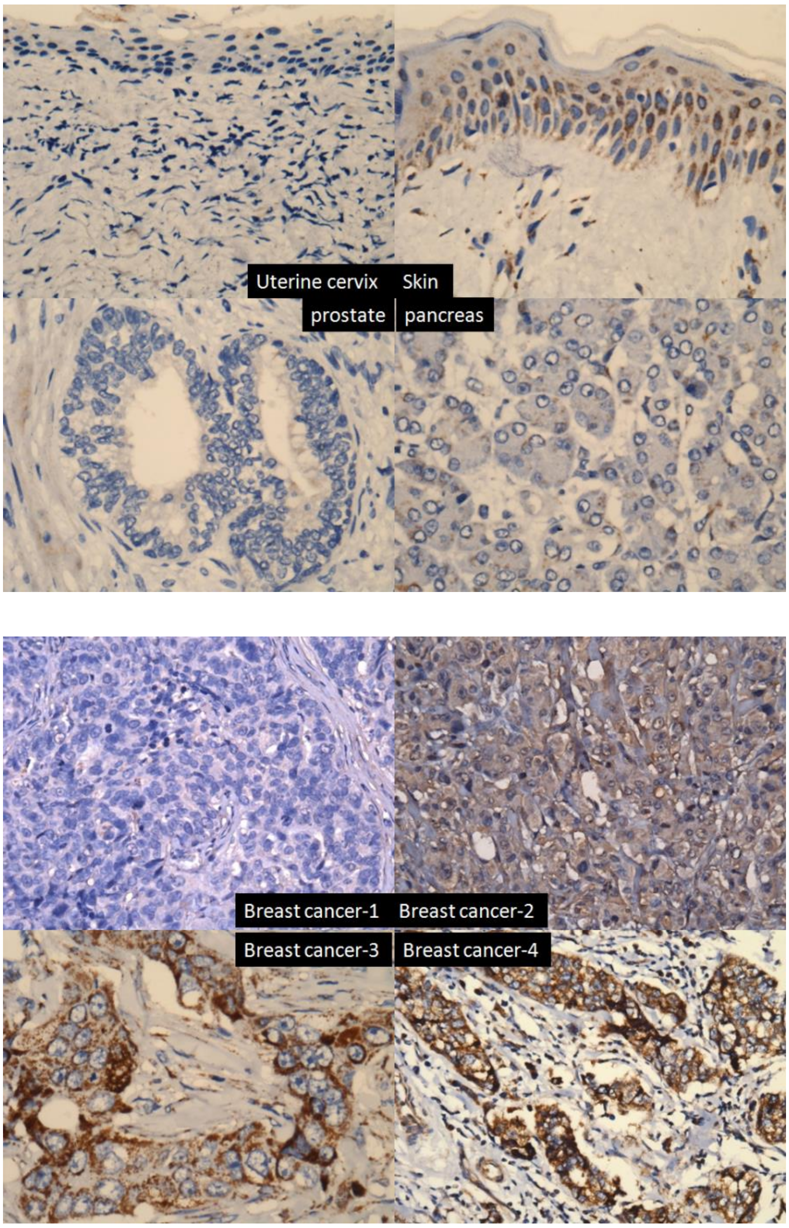

Supplement: Supplementary file 1 — Supplementary information. [file 41598_2020_64950_MOESM1_ESM.pdf]
